# Respiratory sinus arrhythmia during biofeedback is linked to persistent improvements in attention, short-term memory, and positive self-referential episodic memory

**DOI:** 10.3389/fnins.2022.791498

**Published:** 2022-09-13

**Authors:** Lukas Bögge, Itsaso Colás-Blanco, Pascale Piolino

**Affiliations:** ^1^Laboratoire Mémoire, Cerveau et Cognition, Université Paris Cité, Paris, France; ^2^Institut Universitaire de France (IUF), Paris, France

**Keywords:** heart rate variability biofeedback, respiratory sinus arrhythmia, cardiac vagal control, self-regulation, virtual reality, cognitive training, executive functions, self-referential episodic memory

## Abstract

**Background:**

Heart rate variability (HRV) biofeedback, an intervention based on the voluntary self-regulation of autonomic parameters, has been shown to affect prefrontal brain functioning and improve executive functions. The interest in using HRV biofeedback as cognitive training is typically ascribed to parasympathetic activation and optimized physiological functioning deriving from increased cardiac vagal control. However, the persistence of cognitive effects is poorly studied and their association with biofeedback-evoked autonomic changes has not yet been explored. In addition, no study has so far investigated the influence of HRV biofeedback in adults on long-term episodic memory, which is particularly concerned with self-referential encoding processing.

**Methods:**

In the present study, a novel training system was developed integrating HRV and respiratory biofeedback into an immersive virtual reality environment to enhance training efficacy. Twenty-two young healthy adults were subjected to a blinded randomized placebo-controlled experiment, including six self-regulation training sessions, to evaluate the effect of biofeedback on autonomic and cognitive changes. Cardiac vagal control was assessed before, during, and 5 min after each training session. Executive functions, episodic memory, and the self-referential encoding effect were evaluated 1 week before and after the training program using a set of validated tasks.

**Results:**

Linear mixed-effects models showed that HRV biofeedback greatly stimulated respiratory sinus arrhythmia during and after training. Moreover, it improved the attentional capabilities required for the identification and discrimination of stimuli ($\eta_{\text{p}}^{2}$ = 0.17), auditory short-term memory ($\eta_{\text{p}}^{2}$ = 0.23), and self-referential episodic memory recollection of positive stimuli ($\eta_{\text{p}}^{2}$ = 0.23). Episodic memory outcomes indicated that HRV biofeedback reinforced positive self-reference encoding processing. Cognitive changes were strongly dependent on the level of respiratory sinus arrhythmia evoked during self-regulation training.

**Conclusion:**

The present study provides evidence that biofeedback moderates respiration-related cardiac vagal control, which in turn mediates improvements in several cognitive processes crucial for everyday functioning including episodic memory, that are maintained beyond the training period. The results highlight the interest in HRV biofeedback as an innovative research tool and medication-free therapeutic approach to affect autonomic and neurocognitive functioning. Finally, a neurocognitive model of biofeedback-supported autonomic self-regulation as a scaffolding for episodic memory is proposed.

## Introduction

Heart rate variability biofeedback (HRVB) is a training technique that relies on the self-regulation of autonomous nervous system (ANS) processes to optimize physiological functioning affecting cognition (Lehrer and Gevirtz, 2014). In this context, autonomic activity is indexed by the heart rate variability (HRV), i.e., the periodic change in the cardiac rhythm. HRV characterizes cardiac-brain interactions and has important implications for psychophysiological research. It is a recognized indicator of a person’s physiological and behavioral regulatory capacities underlying physical and psychological resilience (Thayer and Lane, 2000; Andreassi, 2010; Holzman and Bridgett, 2017). Moreover, increased levels of HRV have been associated with beneficial effects for health (Gevirtz, 2013; McCraty and Shaffer, 2015; Lehrer et al., 2020) and cognitive performance (McCraty, 2003, 2016; Hansen et al., 2004; Thayer et al., 2009; Forte et al., 2019). Different theories suggest that the interaction between HRV and cognition is associated with cardiac vagal control (CVC), which is an index of the tonic and phasic parasympathetic activity that the vagus nerves exert to regulate the heart (Lehrer et al., 2000; Thayer and Lane, 2000, 2009; McCraty and Childre, 2010; Shaffer et al., 2014). In this context, the Neurovisceral Integration Model (Thayer and Lane, 2000; Thayer et al., 2009) assumes that a set of cortical-subcortical neural circuits, associated with cardiac as well as cognitive regulation, is modulated *via* an inhibitory pathway linked to the prefrontal cortex that can be interrelated with vagal traffic. Hence, the authors proposed that vagally mediated HRV reflecting CVC is positively correlated with prefrontal cortex performance, which in turn is linked to diverse cognitive functions.

Both the Psychophysiological Coherence model (McCraty and Childre, 2010) and the Resonance Frequency Training model (Lehrer et al., 2000) argue that stronger vagal afferent outflow and prefrontal cortex stimulation can be achieved by optimizing the dynamic structures and interplay of physiological rhythms. According to McCraty and Childre (2010), this optimization is achieved by creating physiological coherence, represented by orderly and stable rhythms, within and between regulatory systems through slow breathing and activation of positive emotions. The Resonance Frequency Training model theorizes that slow breathing at the individual resonance frequency of around one breathing cycle per 10 s (≈0.1 Hz) evokes an alignment of the respiration, heart rate, and blood pressure rhythms reflecting enhanced homeostatic regulation (i.e., baroreflex gain). The techniques involved in the two models both focus on a stable alignment of oscillatory systems driven by vagal influence of the parasympathetic branch (i.e., CVC) that produces auto-coherent (sinusoidal) heart rate waveforms with greater amplitudes. Furthermore, both have in common that HRV is mainly driven by respiration *via* the vagus nerve, also referred to as respiratory sinus arrhythmia (RSA). The latter thus reflects respiration-related CVC which can be assessed by measuring the RSA amplitude (heart rate difference from peak to trough across the breathing cycle) which captures cardiac-respiratory synchrony and HRV amplitude. Another prominent marker of vagally mediated HRV that describes CVC is the root mean square successive difference in the heart period series (RMSSD; Laborde et al., 2017; Shaffer and Ginsberg, 2017). The difference between these two indices is that RSA amplitude reflects CVC which is more tightly linked to respiration and physiological coupling than RMSSD. Accordingly, RSA amplitude is more strongly affected during HRVB and may serve as a more precise indicator of training success whereas RMSSD may better capture overall influences on CVC.

The models presuppose physiological state awareness for voluntary autonomic control. Such awareness can be created through biofeedback, which refers to the measurement and feedback of endogenous physiological parameters. In this context, cardiac and often also respiratory responses, typically presented through the visualization on a screen, are utilized by the user to adapt the HRV regulation strategy (e.g., change in breathing rhythm). Previous HRVB procedures based on the Resonance Frequency Training model or the Psychophysiological Coherence model, also sometimes termed RSA biofeedback, showed positive effects on diverse cognitive functions in healthy adults (see Dessy et al., 2018 and Tinello et al., 2021 for a review), including acute improvements in inhibitory control and attention measured directly after only one session of training (Sherlin et al., 2010; Prinsloo et al., 2011, 2013; Blum et al., 2019) as well as improvements maintained for 1 week in verbal short-term memory, decision making, inhibitory control, and sustained attention (Sutarto et al., 2010, 2012, 2013). In clinical settings large gains in cognitive functions were also achieved, such as improvements in attention, short- and long-term memory (Ginsberg et al., 2010; Lloyd et al., 2010). Despite these first promising results, the relationship between biofeedback-induced changes in the ANS and cognitive improvements as well as the importance of the biofeedback signal remain unclarified. To the best of our knowledge, no previous HRVB study has so far quantified the relationship between autonomic and cognitive changes or included a control placebo condition. Previous findings indicated that when such a condition was included in a respiratory biofeedback study, no positive effect of the biofeedback component could be found (Kapitza et al., 2010; Tinga et al., 2019). Moreover, self-regulation training such as mindfulness and meditation practices are also known to increase HRV amplitude and coherence if they involve slow breathing or the evoking of positive emotions (Cysarz and Büssing, 2005; McCraty and Shaffer, 2015) and may evoke similar levels compared to HRVB interventions (Brinkmann et al., 2020). In addition, none of the previous studies included blinded randomized control trials and an effect maintenance period between the training and post-training cognitive evaluation. Therefore, it remains unclear whether HRVB benefits cognition beyond the training period and whether these cognitive changes were mediated through physiological changes.

In addition, virtual reality (VR), an emerging tool in psychological research, has been used only very rarely for HRVB practices. The interest of an immersive VR-application is that it can introduce new ways in which the user is stimulated and how biofeedback is delivered during training. In this regard, VR can, compared to computer screens, increase the level of immersion, user engagement, sense of embodiment (Kilteni et al., 2012) and presence, i.e., the feeling of being located in and of responding to a virtual environment as if it were real (Fuchs, 2017; Armougum et al., 2019). These properties are particularly useful to increase the intensity of attentional commitment to the biofeedback (Sanchez-Vives and Slater, 2005) and to manipulate the emotional mode (Riva et al., 2007). Both a high level of attention and positive affectivity are crucial for HRVB task success and may positively influence training outcomes. The role of VR for biofeedback purposes to improve cognitive and emotional states supporting training has already been emphasized previously (Cho et al., 2004; Shaw et al., 2007; Repetto et al., 2009; Shiri et al., 2013; Gromala et al., 2015; Blum et al., 2019, 2020; Rockstroh et al., 2019, 2020). Furthermore, a stronger sense of presence has been associated with greater skill transfer to real life applications (Sanchez-Vives and Slater, 2005; Grassini and Laumann, 2020), highlighting the benefits of VR for biofeedback interventions.

The relevance of HRVB for cognition has been studied primarily for prefrontal cognitive functions involved in early stage information processing but never for long-term memory in adults and self-referential processing in general. Episodic memory (EM) is a unique memory system that records specific events experienced by oneself (Tulving, 1985, 2002). Self-focused experience during encoding is critical in determining the idiosyncratic nature of long-term memory (Hommel, 2004; Bergouignan et al., 2014; Makowski et al., 2017; Bréchet et al., 2018). Several paradigms have been developed to explore the role of the self in EM performance. The best-known paradigm for studying this relationship is self-reference manipulation (Rogers et al., 1977; Symons and Johnson, 1997; Klein, 2012). Self-reference processing consists in linking new to-be-remembered information to the self, either *via* its narrative component (e.g., pre-stored self-knowledge and autobiographical memories) or its minimal component (e.g., the “I” who is experiencing “here and now” or the body self) (Gallagher, 2000), that provides a memory advantage to the new information known as the self-reference effect (SRE). Firstly, self-regulation training requires attentional focus to the self which is assumed to elicit self-awareness and self-reference processes (Vago and David, 2012). The self also extends to the embodied self, namely the virtual representation of the person’s own body and physiological functions (Monti et al., 2019). In this regard, biofeedback may further support the stimulation of self-referential processing as it provides access to, and facilitates the monitoring of, self-related processes that normally go unnoticed (e.g., changes in the heart rate). Secondly, self-referential processing is characterized by a greater activation of the ventromedial prefrontal cortex (vmPFC; Northoff et al., 2006; D’Argembeau et al., 2007; Denny et al., 2012; Martinelli et al., 2013; Yaoi et al., 2015), a cortical area that is also involved in cognitive and physiological self-regulation processes (Hänsel and von Känel, 2008; Thayer et al., 2009; Maier and Hare, 2017). Initial evidence that HRVB alters brain functioning related to self-processing after training has been recently reported (Schumann et al., 2021; Bachman et al., 2022). Thus, we assume that VR-HRVB training may alter the mnemonic properties of self-reference through self-focused attention and ANS-mediated changes in the neurophysiological functioning related to the vmPFC.

The target of this study was to evaluate the lasting impact of increasing CVC during HRVB on the task performance of a wide range of cognitive functions in young healthy adults, including attention and executive functions, and for the first time, long-term EM and self-referential processes. The work investigated the specific effect of biofeedback during HRV self-regulation training on cognition as well the association between autonomic and cognitive changes. A novel system was developed coupling real-time biofeedback with immersive VR. Two randomized and blind groups were compared including a control placebo condition that differed from the HRVB intervention group by the absence of biofeedback. It was hypothesized that (1) young healthy adults practicing HRV self-regulation training stimulate more profoundly CVC indexed by RSA amplitude and RMSSD during training when biofeedback is provided with greater effects on RSA amplitude, (2) HRVB training sustainably improves executive and emotional control as well as EM compared to training without biofeedback, (3) increases in EM performance are greater for items encoded with self-reference due to changes in the SRE, and (4) improvements in behavioral functions are positively correlated with the level of respiration-linked CVC (i.e., RSA amplitude) during training.

## Materials and methods

As part of this work, a public data repository (Bögge et al., 2021) was created that contains in addition to the datasets, the digital materials used and on materials and methods.

### Participants and study design

A minimum sample size of 20 participants for the cognitive effects of HRVB was estimated before data collection based on the only available publications with similar protocols that also included a cognitive maintenance period and a control group (Sutarto et al., 2010, 2012, 2013). In this line, Sutarto et al. (2010) found significant group-by-time interaction effects on memory and attention using a sample of only 16 subjects. Furthermore, partial eta-square values greater than 0.16 were determined for group-by-test interaction effects on memory and attention measures reported by Sutarto et al. (2012), who replicated their previous study with a bigger sample size, by calculating the group difference between time effect sizes. Based on the assumption that partial eta-square values greater than 0.10 will be observed for interaction effects in this study, a minimum sample size of 20 participants was calculated using G*Power 3.1.9.4 (Faul et al., 2009) with alpha = 0.05, power = 0.80, and correlation among repeated measures = 0.5. For the physiological measures a minimum sample size of 90 (15 participants × 6 repeated measures) was determined with alpha = 0.05 and power = 0.80. The corresponding power analysis was performed for an independent *t*-test with nested data (repeated measures per participant). One sample corresponds to the measure of a single session. Each participant had to pass six training sessions. Therefore, 90 samples correspond to the sum of all sessions of 15 participants (6 sessions × 15 participants). We assumed moderate-to-strong group differences (Cohen’s *d* > 0.6) as HRVB affects CVC very strongly in young healthy adults (Lehrer et al., 2003; Prinsloo et al., 2013; Blum et al., 2019; Rockstroh et al., 2019) whereas effects in the control group were expected to be at best modest (Krygier et al., 2013; Léonard et al., 2019).

Participants were recruited through an online application form that was accessible *via* an online platform communicating experiment offers and hangouts in the university building. In total, 25 young healthy adults were enlisted for a series of six self-regulation training sessions that was preceded and followed by a cognitive assessment session. All the following inclusion and exclusion criteria in this section were established prior to data collection. The study included only 20- to 30-year-old native French speakers who reported not being concerned by any of the following conditions: history or treatment for psychiatric, neurological, cardiac, or severe respiratory disorders, treatment with a cognitive impact, chronic pain, frequent dizziness or nausea, vertigo, impaired but not corrected visual or auditive capabilities, substance abuse. Further, participants were required to have completed at least 12 years of education and be novices in meditation, as regular mindfulness practice could impact self-regulation capabilities. Finally, the analysis took account only of participants who attended each session and whose test scores (see below) did not indicate high levels of anxiety or depression. After enrollment, participants were divided pseudo-randomly into a biofeedback group (BG) and an active control group (CG). In total 22 subjects were included for the data analysis, of which 12 were in the BG (8 women, mean age 24 ± 2.48 years) and 10 in the CG (6 women, mean age 26.88 ± 3.93 years). Three participants were excluded because two exhibited high anxiety scores and one did not show up for the last assessment session.

Experiments were conducted individually between April 2019 and March 2020 at the Institute of Psychology of the Université Paris Cité, with participants being blind to the group assignment and existence of two different experimental conditions (with and without biofeedback). Informed and written consent was obtained at the beginning of the first session. At the end of the last session participants were compensated with a gift voucher. All procedures performed in studies involving human participants were in compliance with the ethical standards according to institutional guidelines and national legislation (Jardé law, 2016). Data privacy protection, collection, and processing were in accordance with the 2018 reform of Eu data protection rules (2018).

Cognitive evaluations and training were conducted in two different experimental rooms. To keep the instructions and information given equal between groups and to circumvent differences in subject-expectancy, it was mentioned to each participant during recruitment and at the beginning of the first assessment that training might improve health as well as cognitive functions in general. Demographic details, the new Body Mass Index (BMI) for the measure of body fat, and hours of sport per week were recorded at the beginning of the first session. Furthermore, all participants reported being right-handed and having no prior experience of biofeedback training. Levels of anxiety and depression were assessed at the beginning of the first and last session with the score-based validated French version of the State-Trait Anxiety Inventory (Spielberger, 1983; Schweitzer and Paulhan, 1990) and the shortened and validated French version of the Beck Depression Inventory (Beck and Beamesderfer, 1974; Collet and Cottraux, 1986). According to the manual of the State-Trait Anxiety Inventory and Beck Depression Inventory, the severity of the syndromes was classified as high when scores were above 55 and 15, respectively. Measures of participant characteristics did not significantly differ at a significance level of alpha = 0.05 between groups except for the BMI (Table 1). It was therefore added as a control variable in the HRV analysis. Anxiety and depression scores did not change from pre- to post-test and did not differ between groups at post-test (*ps* > 0.527). Further, participants were asked to refrain from smoking and consuming caffeinated or alcoholic substances during training days until the end of the sessions. After the end of the study, participants had 1 week in which to complete, if they wished, an anonymized online follow-up questionnaire that assessed their subjective feeling about training effects on their cognition and wellbeing.

**TABLE 1:** Participant characteristics.

Control variable	Mean (*SD*)		Group effect	
	BG (*n* = 12)	CG (*n* = 10)	*F* (1,20)	*p*
Proportion of females^a^	66%	60%	0.11	*0.746*
Proportion of subjects regularly playing video games^a^	34%	20%	0.49	0.484
Current Years of Education	14.17 (1.70)	14.50 (1.51)	0.23	0.635
BDI (Depression)	3.67 (3.70)	4.50 (3.92)	0.26	0.614
STAI-Y1 (State Anxiety)	30.67 (8.29)	28.50 (6.72)	0.44	0.515
STAI-Y2 (Trait Anxiety)	35.08 (9.19)	34.58 (7.76)	0.03	0.874
Hours of sport per week^b^	2.00 (2.25)	2.00 (2.5)	236	0.932
New Body Mass Index (BMI)^b^	20.85 (2.30)	23.64 (7.80)	132	0.011

BG, biofeedback group; CG, active control group; BDI, beck depression inventory; STAI, state trait anxiety inventory.

^a^Group effect is represented by X^2^(1, N = 22).

^b^Due to violation of normality distribution the median, inter-quartile-range (in parenthesis) and results from the Mann-Whitney-Wilcoxon test (U[24,20]) are reported.

### Training system

**FIGURE 1:**
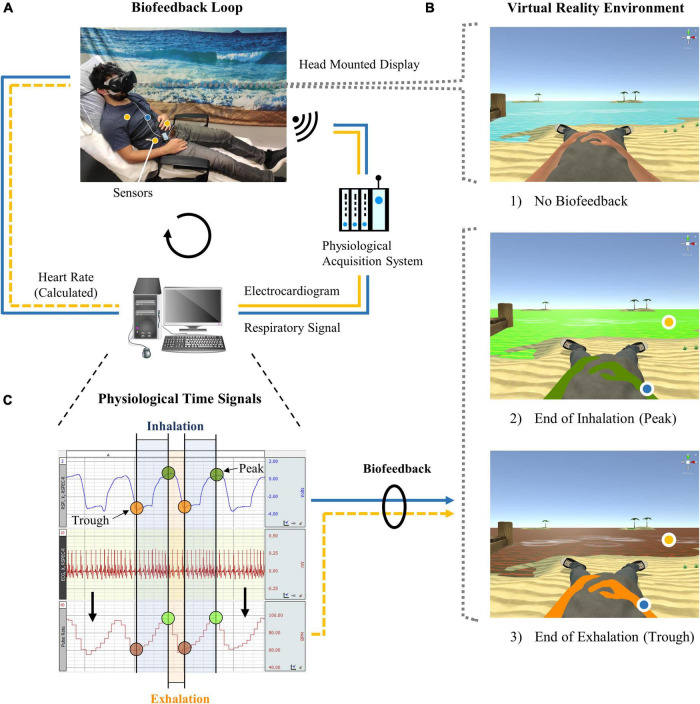
Virtual reality biofeedback system. **(A)** During heart rate variability biofeedback training, which involved slow and rhythmic breathing, abdominal and thoracic circumference as well as an electrocardiogram were recorded through a respiration belt and surface electrodes (hidden under the shirt). Signals were processed by a computer and could be visualized in the virtual reality environment. **(B)** Participants in (1) the active control group followed the same training but received no biofeedback. Biofeedback was displayed by colors of the avatar (respiration) and sea (heart rate) that changed gradually from (2) green to (3) orange/red during exhalation or when the heart rate dropped, respectively, and vice versa. **(C)** The target of the training was, as exemplified here, to maintain synchrony between the respiration and heart rate time signals (i.e., similar color between the avatar and sea) and to increase the heart rate amplitude (i.e., stronger shades of green and red for the sea).

A new VR biofeedback system was developed for the self-regulation training of HRV. The novelty compared to conventional HRVB training systems is that cardiac as well as respiratory responses are embodied in an immersive VR environment displayed *via* a head mounted display (Vive; HTC and Valve, 2015; Figure 1). In this virtual world, created with the computer software Unity 3D (Unity Technologies, 2018), the user takes on the form of a human avatar (first-person body view) half-reclining on a lonely beach gazing at the open sea. Changes in thoracic or abdominal circumference and an electrocardiogram (ECG) are recorded wirelessly by the BIOPAC data acquisition system MP150 (Biopac Systems, Inc., n.d.a,b,c,d,e,f) including surface electrodes and a respiratory belt transducer. Signals are then processed by customized scripts embedded in Unity 3D that allow the data stream to be coupled with the VR environment. In this way, HRV and respiration can be embodied in real time by the change of color of the sea and the avatar, respectively. Consequently, the visual biofeedback can be used in combination with a training method, e.g., a breathing exercise, to regulate the heart and respiratory functions in a targeted manner. A more detailed account of the setup as well as of the following training procedure is provided in the public data repository (Bögge et al., 2021).

### Training procedure and physiological recordings

Each participant of the BG and CG attended individually six training sessions over a period of 3 weeks. For each week, two sessions had to be scheduled on different days that lasted around 1 h including 25 min of training in VR. Participants of both groups followed the same self-regulation training (i.e., regulation of the respiration and heart rate) specified below during which only the BG received biofeedback. Physiological parameters were recorded each session following guidelines of the manufacturer’s application note 233 (Findlay and Dimov, 2016). For the HRV study design, processing, analysis, and report we followed the guidelines of the Task Force of the European Society of Cardiology and the North American Society of Pacing and Electrophysiology (1996) and recommendations for HRV in psychophysiological research (Laborde et al., 2017).

At the beginning of each session participants were seated on a reclining chair where they remained seated throughout the session in a Fowler position with a whole trunk inclination at 45° and knees slightly bent. Both groups were provided with similar instruction sheets that included information about the principles of HRV, RSA, Resonance Frequency Training, mindfulness, meditation, and HRV training techniques (see repository Bögge et al., 2021). Only the BG received extra specifications for the biofeedback signals. The given objective was the same for both groups, i.e., to increase levels of HRV and synchrony between heartbeat and respiratory oscillations. To ensure that the participants felt at ease and complied with the instructions, they were also orally briefed and debriefed before and after each training session, respectively, with questions being clarified. Additionally, instructions were recapitulated during a 5-min habituation period in VR prior to starting training in the first session. During this period, subjects were free to explore and familiarize themselves with the VR environment and biofeedback. Otherwise, participants were immersed in VR only during the training phase. For the duration of the training, participants were instructed to place their hands on their belly to mimic the posture of their avatar, to restrict their movements except for the head, and to refrain from talking.

Self-regulation training was based on the Resonance Frequency Training protocol of Lehrer et al. (2000). Participants were asked to breathe rhythmically at around one cycle per 10 s, inhaling diaphragmatically and exhaling through pursed lips at equal intervals. Shallow and natural inhalation was advised to prevent hyperventilation. No pacer was provided; instead, participants of both groups were instructed to adjust the respiratory rate that elicited the largest heart rate oscillations. However, for the first 2 min of each training session, the rhythm (i.e., 1 cycle per 10 s) was dictated to help the subjects get a better sense of timing. Moreover, in order to facilitate the monitoring and control of the HRV, participants were instructed to conduct a body scan (Ditto et al., 2006), a common meditation practice involving the focalization of attention on the sensations of respiration and heartbeat in a certain part of the body. Attention had to be directed to five different body parts successively for 5 min each. The only difference between groups was that the BG received visual biofeedback. Consequently, they were free to choose whether to concentrate on body sensations or on the colors in the VR environment for information on their own HRV, whereas the CG had to rely solely on body perception. For the BG, the body part displaying biofeedback changed automatically and indicated the locus on which the user should focus. Contrary, the CG was instructed orally to shift their attention each 5 min. During training, a running ventilator was placed 1.5 m in front of the participant and sea sounds were played to increase feelings of immersion. Furthermore, the physiological recordings included two 5-min resting-state measures to determine the baseline and recovery level; one shortly before the VR immersion and one starting 5 min after the training. In addition, the participant and experimenter were separated by blinds during recordings to reduce distractions and increase levels of comfort.

### System assessment

In order to explore VR and attention related characteristics of the present system, self-report evaluations of a subsample of 16 Participants (9 in the BG) were recorded. Participants were asked to rate their sense of presence and embodiment (i.e., body ownership, agency, location of the body, and external appearance) in the VR after each second training session using adapted versions of the Slater-Usoh-Steed questionnaire (Slater and Steed, 2000; Usoh et al., 2000) and the embodiment questionnaire proposed by Gonzalez-Franco and Peck (2018), respectively. Participants were assessed either at session 1, 3 and 5 (A) or at session 2, 4 and 6 (B). The assessment sequence A or B was randomized among participants. Half of the participants were assessed in sequence A (5 in the BG) and the other half in sequence B (4 in the BG). Furthermore, subjects were asked after each training session to estimate the amount of time they felt attentive to the task and the amount of time they felt drowsy during self-regulation training (see repository Bögge et al., 2021). In addition, subjects reported the level of fatigue they were feeling at the moment before and after each session. This item was based on the Right Now item from the Brief Fatigue Inventory (Mendoza et al., 1999) with “problems thinking clearly” added so that mental fatigue was also reflected in the score (Guidelines, 1998). Recordings of one session in the CG had to be excluded due to technical problems. In total there were 47 samples for the presence and embodiment scales each and 95 samples for the attention and fatigue scores each.

### Cognitive assessment

**FIGURE 2:**
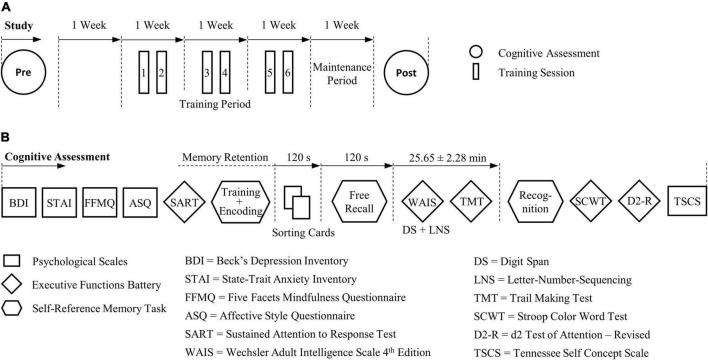
Course of study and procedure of the cognitive pre- and post-assessment. **(A)** Cognitive effects that were sustained 1 week after self-regulation training were assessed. **(B)** The experimental procedure was identical between groups and tests. For the self-reference episodic memory task parallel task versions were devised for the pre- and post-test, respectively.

Standard and validated tests and scales including measures of memory, executive and affective control, mindfulness, and self-concept were administered with a pretest-posttest experimental design. Participants were individually assessed with an identical procedure between tests and groups around 1 week before and around 1 week after the training period (Figure 2). Pre- and post-assessments were carried out by two different experimenters, with the posttest being performed blind.

#### Psychological scales

At the beginning of each assessment, participants were asked to complete four computerized self-administered questionnaires, including the Beck Depression Inventory (Beck and Beamesderfer, 1974; Collet and Cottraux, 1986) and State-Trait Anxiety Inventory (Spielberger, 1983; Schweitzer and Paulhan, 1990) that were used for screening (see 2.1). Additionally, the state of mindfulness and emotional regulation capabilities were assessed using the French translation of the Five Facet Mindfulness Questionnaire (Baer et al., 2006; Heeren et al., 2011) and a shortened and validated French version of the Affective Style Questionnaire (Hofmann and Kashdan, 2010; Makowski et al., 2018), respectively. Questionnaires were administered and scores calculated with the Python module Neuropsydia (Makowski and Dutriaux, 2016) in WinPython64 version 3.7.2 (Raybaut and 2014-2019+ The WinPython Development Team, 2019) based on Python version 3.7. Assessments were concluded with a self-assessment scale of the subjective perception of the self-concept by means of three shortened French versions of the Tennessee Self-Concept Scale (Fitts and Warren, 1996; Duval et al., 2007; Compère et al., 2018). While all three forms comprised the same 21 items of the original Tennessee Self-Concept Scale, they evaluated the self-concept in different time dimensions (i.e., past, present, future). Global scores were retrieved for each form with higher scores indicating a more positive self-perception.

#### Executive functions

Executive functions including sustained attention, processing speed, cognitive flexibility, inhibition, as well as short-term and working memory were evaluated by means of six cognitive tests (Figure 2). Sustained attention and inhibitory control were checked before the memory task by a computerized version of the Sustained Attention to Response Task (Robertson et al., 1997; Makowski and Dutriaux, 2016) which is a Go/No-Go task with a majority of Go stimuli (89%). The mean reaction time as well as the rate of correct Go and No-Go responses were determined. Attention and concentration capabilities were also evaluated at the end of the assessment by the French revised version of the d2 Test of Attention (Brickenkamp et al., 2015). Norm-referenced scores of the Processed Target Objects, Percentage of Errors (or Error Rate) and the Concentration Performance were derived. Cognitive interference involving inhibition processes was investigated using the Stroop Color and Word Test (Stroop, 1935). In line with recommendations of Scarpina and Tagini (2017), a new global score was introduced, representing the interference effect that incorporated the processing time and the corrected as well as uncorrected errors of each subtest (see repository Bögge et al., 2021). Further, abilities of cognitive flexibility were assessed by the Trail Making Test (Reitan, 1958). For the analysis, the completion time of part A and part B and the difference between the Trail Making Test parts (B-A) in seconds were included. For the Stroop Color and Word Test and Trail Making Test the French translations from the GREFEX test battery (Godefroy et al., 2010) were used. Working memory capabilities were assessed by the Digit Span and Letter-Number Sequencing tests from the French translation of the fourth edition of the Wechsler Adult Intelligence Scale (Wechsler, 2010) that tested the immediate recall of series of digits and letters. Norm scores were derived for all digit spans in the Digit Span (i.e., forward, backward, sequencing, and total) and Letter-Number Sequencing tests as well as for the Working Memory Index.

#### Self-reference episodic memory task

**FIGURE 3:**
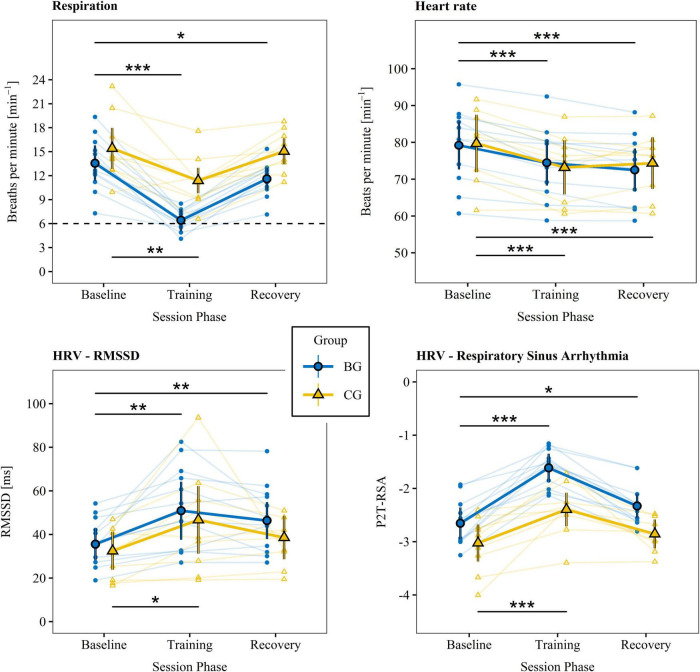
Physiological measures. Physiological parameters were recorded directly before (baseline), during (training), and 5 min after (recovery) each training session. The small data points represent means of individual participants summarized over training sessions. The large data points reflect estimated marginal means summarized across participants and sessions that account for random subject effects and individual differences in the new Body Mass Index. Vertical lines indicate 95% CIs. The dashed line stands for the target breathing rate during training. Phases were compared between baseline and training as well as baseline and recovery for the BG (top lines) and CG (bottom lines), respectively. HRV, heart rate variability; RMSSD, root mean square of successive differences; BG, biofeedback group; CG, active control group; P2T-RSA, natural logarithm of the respiratory sinus arrhythmia calculated by the peak-to-trough method. ***p < 0.001. **p < 0.01. *p < 0.05.

To test EM, in particular recollection memory, involving self-reference processes, we adhered to validated protocols that investigated the mnemonic SRE in healthy young and older subjects and in pathological condition (Lalanne et al., 2013; Compère et al., 2016). All materials, stimuli, and the procedure used for this task derived from the latter two studies which assessed EM using the Remember/Know/Guess (R/K/G) paradigm (Gardiner et al., 2002). Memory probes (see repository Bögge et al., 2021) were personality trait adjectives (e.g., “optimistic” and “malicious”) originating from Anderson’s personality-trait word list (Anderson, 1968). The number of positive and negative items was balanced within and among all lists. Two parallel task versions were used for the pre- and post-test with an identical procedure and different word lists. The task was divided into three phases: (1) encoding, (2) free recall, and (3) recognition (Figure 3).

##### Encoding

Following a short familiarization phase, participants were presented item by item with 48 adjectives of four different conditions which they were instructed to memorize. For every adjective, participants had to make a yes-or-no decision to decide, depending on the condition, whether (a) its first and last letters were in alphabetical order (perceptive condition), (b) it was considered as socially desirable in general (semantic condition), (c) it described them (semantic self-reference condition), or (d) it could be associated to a memory of a personal event (episodic self-reference condition).

##### Free recall

After a short memory retention period they were asked to retrieve without any aid as many adjectives as possible within 2 min. Subsequently, memory was further retained for a second period of around 25 min during which the memory task was interrupted by part of the executive functions test battery (Figure 3).

##### Recognition

Participants were presented with a list of 48 adjectives, half of which (24 items) derived from the encoding conditions while the other half were novel distractor items. The content of the recognition lists was equal between participants and in random order. For each word the participants had to decide whether they had seen it during encoding or not. If the answer was “yes,” an additional question addressed the presence of memory recollection using the R/K/G paradigm (Gardiner et al., 2002). In this line, the participants had to decide whether they remembered (R) in which context the word occurred (they could mentally experience the encoding again), or whether they knew (K) with certainty that the adjective appeared before, but they did not remember encoding details, or whether they guessed (G) its previous presence. Each R response was checked by asking the participant to indicate the corresponding condition.

Recordings included the response decisions and response reaction time during the encoding and recognition phase as well as the adjectives recalled. Measures of memory performance were the proportions of correct free recalls, subjective remembering (correct R responses), and objective source recollections (R responses correctly associated with the encoding condition) among the studied items. Furthermore, the performance of discriminating studied from distractor items was determined by calculating the sensitivity index d-prime (or *d*′). All measures were retrieved in dependence of the encoding condition and the valence of the adjectives.

### Data processing of physiological recordings

Physiological recordings from the training sessions were preprocessed semi-automatically to generate measures of HRV and RSA in accordance with established guidelines, norms, and metrics (Task Force of the European Society of Cardiology and the North American Society of Pacing and Electrophysiology, 1996; Laborde et al., 2017; Shaffer and Ginsberg, 2017). Recordings of the 25-min training phase were split into five non-overlapping 5-min segments per session to match the length with the baseline and recovery phases. Segments of the baseline, training, and recovery phases were then processed and analyzed individually using an adapted version of the Python package NeuroKit2 (Makowski et al., 2020). Based on recommendations by BIOPAC (Biopac Systems, Inc., n.d.f) and the application note 233 (Findlay and Dimov, 2016), preprocessing included a bandpass filter of 0.5–35 Hz for the ECG and a bandpass filter of 0.025–1 Hz for the respiratory signal. In the ECG signal QRS complexes were automatically identified and the timing of the R-peaks within each QRS complex was determined following the *NeuroKit* method. QRS complexes that were shorter than 0.4 or longer than 1.25 times the median of all QRS intervals were rejected. Additionally, any R-peak that occurred less than 500 ms after the preceding R-peak was excluded (i.e., maximum threshold of 120 Hz between two heartbeats). The timing of R-peaks and therefore the heart period series (i.e., R-R intervals) was then corrected based on the algorithm of Lipponen and Tarvainen (2019). The algorithm identified R-peaks that were missing, superfluous, or misplaced, causing very rapid changes in successive R-R intervals that are typical of ectopic beats. In the correction phase, R-peaks were then respectively either deleted, inserted, or moved to the center between the directly adjacent R-peaks. R-peak correction was iterated so that R-peaks were corrected one-by-one starting from the beginning of the time series. To handle the correction of multiple adjacent R-peaks that were missed (e.g., rejection of R-peaks due to movement artifacts) the number of missing R-peaks was estimated whenever a missing R-peak was detected. Equal R-R intervals between inserted and adjacent R-peaks were assumed (i.e., constant heart rate) that were no longer than the 95th percentile plus the standard deviation of R-R intervals in the complete time series. The number of inserted R-peaks was increased until this threshold was undershot. For further details please consult the NeuroKit2 documentation (Makowski, 2021). Raw signals as well as graphical outputs generated from the analysis (e.g., original and corrected RR period, heart rate, and respiration time series) were sighted individually for measurement errors, artifacts, and ectopic beats. The prevalence of ectopic beats in the general adult population is 1–4% and at the median they amount to around 0.01% of all heartbeats (Simpson et al., 2017; Marcus, 2020). It is normal, however, for some humans to experience higher numbers of ectopic beats; excessive numbers are generally defined as >10% of all heartbeats.

Complete sessions were discarded if one or more segments met one of the following exclusion criteria that were established prior to data collection: (1) the raw signal was strongly artifacted or exhibited a high number of ectopic beats (i.e., proportion to total beats >2% or occurrence of more than two consecutive ectopic beats), (2) the analysis repeatedly failed to correct artifacts (e.g., large and abrupt changes visible in the heart period after correction), or (3) respiration cycles could not be properly detected. Recordings of 119 from a total of 132 training sessions were included for further statistical analysis. Eight sessions were excluded because too many ectopic beats were detected, four due to technical problems during data saving, and one because of the occurrence of large movement artifacts.

Measures of HRV were calculated for each 5-min segment. Time domain variables were based on time intervals between normal successive heartbeats (NN). Those included the square root of the mean of the squares of the successive differences between adjacent NNs (RMSSD), the standard deviation of the successive differences between adjacent NNs (SDNN) and the percentage of number of pairs of successive NNs that differed by more than 50 ms (pNN50). In the frequency domain, power spectral density was calculated for three frequency ranges: very low frequency (<0.04 Hz), low frequency (0.04–0.15 Hz), and high frequency (0.15–0.40 Hz). Spectral measures included the natural logarithm of absolute low frequency and high frequency power (lnLF and lnHF) as well as their normalized values. Further variables determined were the absolute power ratio of low frequency to high frequency and ln-transformed measures of the RSA using the Peak-to-Trough (P2T-RSA) and Porges-Bohrer (PB-RSA) method (Lewis et al., 2012).

### Statistical analyses

The statistical analysis was carried out with R 3.6.2 (R Core Team, 2019) using the rstatix (v0.5.0; Kassambara, 2020), lmerTest (v3.1–2; Kuznetsova et al., 2017), and emmeans (v1.4.6; Lenth, 2020) packages. The analyses were performed separately for each dependent variable (DV). Statistical assumptions were checked according to instructions proposed by Kassambara (2019). Linear regression models including within-subject factors were developed based on mixed effect models including random subject effects (blocking factor). These models accounted for random intercepts and slopes along repeated measures of time. Main and interaction effects were calculated from the analysis of variance (ANOVA) or analysis of covariance when covariates were included. Effects of Type 3 are reported here due to the imbalance in group sizes. Following recommendations of Luke (2017) that produce most optimal Type 1 error rates for multiple factors mixed effect models, especially for smaller and unbalanced sample sizes (12–24 subjects), degrees of freedom were calculated using the Kenward-Roger method and unbiased estimates of variance and covariance parameters were determined using the restricted maximum likelihood. Factor effect sizes were based on partial eta-square values and interpreted as small, medium, and large for values of 0.01, 0.06, and 0.14, respectively (Cohen, 1988). Further, group and test comparisons were always concerned with pre-to-post-test and CG-to-BG differences. Estimated marginal means (EMMs) were computed and compared using the emmeans package (v1.4.6; Lenth, 2020). Effect sizes derived from EMMs pairs were based on the parameter *d*_*m*_ which is determined analogously to Cohen’s *d*. Values of 0.25, 0.5, and 0.85 were interpreted, based on recommendations of a HRV distribution analysis (Quintana, 2016), as small, medium, and large, for measures of HRV. EMMs and effect sizes are reported along with the 95% CI. Monotonic correlations were based on Spearman’s rank-order correlation. All significance tests were based on alpha = 0.05 and were two-tailed.

Repeated measures from the training phase were averaged within each session to balance the number of samples between the baseline, training, and recovery phase. Linear regression models were devised with group (BG, CG) as between-subject factor and session phase (baseline, training, recovery) as within-subject factor. Since group means of the BMI differed significantly (see 2.1), the mean-centered BMI was added as a covariate for cardiac measures [DV ∼ new BMI + group * session phase + (1 + session phase | subject)]. EMMs were identified in each group for each session phase. EMMs of the training and recovery measures were then compared with the baseline within groups. Additionally, EMMs for the training and recovery phase were compared between groups using a model that adjusted means for baseline values (DV ∼ baseline + group * session phase).

Behavioral outcomes were each analyzed with a factorial design including the between-subject factor group (BG, CG) and within-subject factor test (pre, post). Average values and group × test interaction effects were determined for the measures apart from the EM task (DV ∼ group * test). EM task variables additionally included the encoding condition (perceptive, semantic, semantic self-reference, episodic self-reference) and valence (positive, negative) as fixed factors. Memory performance scores were controlled for the number of responses and reaction time during encoding. Effects involving the interaction of group and test were checked for every encoding condition separately. If significance was observed for any effect in any condition, the variable was added as a covariate to the following analysis of memory responses. Likewise, effects on the memory retention period between the encoding and recognition phase were tested by means of 2 (BG, CG) × 2 (pre, post) ANOVA. Since we were interested in EM performance in both reference conditions (self, non-self), regression models were devised separately for items with self-reference (semantic self-reference and episodic self-reference conditions) and without self-reference (perceptive and semantic conditions) [DV ∼ (covariate) + group * test * encoding condition * valence + (1 + test | subject)]. Effects involving group × test interaction and EMMs for each group-test combination were determined. Moreover, differences between reference conditions were characterized by the calculation of the SRE. The latter can be described by the difference in memory performance for items encoded with self-reference compared to items encoded in general semantic context (Glisky and Marquine, 2009; Lalanne et al., 2013; Compère et al., 2016). To this effect, group × test × valence interaction effects were identified for encoding condition differences (semantic-to-semantic-self and semantic-to-episodic-self). Where a statistically significant involvement of the encoding condition or valence was detected, group × test interaction effects on each level of the respective variable were determined. For all significant group × test interaction effects, EMMs or average scores were compared between pre- and post-recordings. Furthermore, the effect of the other cognitive measures on significant memory outcomes was controlled for. To do so, variables that exhibited a significant group × test interaction effect were added separately as covariates to the regression model. Finally, to study the relationship between physiological and behavioral results on a participant level, a correlation analysis was conducted for every behavioral variable. Pre-to-post differences of each participant were correlated with the baseline-adjusted EMM of P2T-RSA of the training. The P2T-RSA was chosen as the main indicator of vagally mediated HRV because it better captures vagal influences on a wide range of breathing frequencies compared to lnHF and lnLF and is less disturbed by sympathetic influences than the RMSSD. For significant correlations, the P2T-RSA × test interaction effect (groups combined) was determined [DV ∼ (covariate) + P2T-RSA * test (1 | subject)].

## Results

### System assessment

Separate mixed model analyses including random subject effects revealed no group differences for the sense of presence, sense of embodiment, or for any of its subcategories. However, compared to the CG the BG felt more attentive, *t*(14) = 3.89, *p* = 0.002, *d*_*m*_ = 1.01, and less drowsy, *t*(14.1) = −3.41, *p* = 0.004, *d*_*m*_ = −0.53, during the training phase. Self-reported fatigue did not differ between groups before, but after training, *t*(14.2) = −2.39, *p* = 0.031, *d*_*m*_ = −0.73. Only the CG felt significantly more fatigue after training. Further details are provided in the Supplementary material A Table A1.

### Maintenance and retention periods

In average 6.50 (*SD* = 1.83) and 6.20 (*SD* = 2.74) days passed between the last training session and post assessment in the BG and CG, respectively. No differences were found between group means, *t*(20) = 0.31, *p* = 0.762, *d* = 0.13. In the BG the retention period from encoding to the recognition phase was in average 27.08 (*SD* = 2.49) min during pre-assessment and 24.58 (*SD* = 1.16) min during post-assessment, while in the CG it was 26.05 (*SD* = 2.05) and 24.80 (*SD* = 2.49) min, respectively. A significant main effect of test was identified, *F*(1,20) = 8.79, *p* = 0.008,$\,\eta_{p}^{2}$ = 0.19, because participants performed the tests during the retention period more rapidly in the post-assessment. No significant group effect, *F*(1,20) = 0.407, *p* = 0.531, $\eta_{p}^{2}$ = 0.01, nor interaction effect *F*(1,20) = 0.976, *p* = 0.335, $\eta_{p}^{2}$ = 0.02, was observed.

### Physiological measures

Estimated marginal means derived from linear mixed models of the respiration rate, heart rate, and primary HRV variables indexing CVC and training success (i.e., RMSSD and P2T-RSA; Laborde et al., 2017; Shaffer and Ginsberg, 2017) as well as statistics of the within- and between group comparisons are given in Tables 2, 3 and are illustrated in Figure 3. The supplementary indices are presented in the Supplementary material B (Supplementary Tables B1, B2). There were no significant group differences between baseline measures for any of the physiological parameters.

**TABLE 2:** Within-group comparisons of physiological measures.

Measure	Biofeedback group							Active control group						
	Baseline (*n* = 68)	Training (*n* = 68)	Recovery (*n* = 68)	Training-baseline		Recovery-baseline		Baseline (*n* = 51)	Training (*n* = 51)	Recovery (*n* = 51)	Training-baseline		Recovery-baseline	
						
	*EMM*	*EMM*	*EMM*	*t*(19.0), *p*	Effect size *d*_*m*_	*t*(19.0), *p*	*Effect* size *d*_*m*_	*EMM*	*EMM*	*EMM*	*t*(19.0), *p*	Effect size *d*_*m*_	*t*(18.9), *p*	Effect size *d*_*m*_
Respiration rate [min^–1^]	13.56 [11.34, 15.78]	6.42 [5.04, 7.80]	11.59 [10.18, 13.01]	−7.33, **<0.001**	−2.89 [−4.48, −1.31]	−2.79, **0.012**	−0.80 [−1.50, −0.09]	15.41 [12.84, 17.98]	11.38 [9.79, 12.97]	15.05 [13.42, 16.68]	−3.58, **0.002**	−1.63 [−2.86, −0.41]	−0.45, 0.659	−0.15 [−0.84, 0.55]
Heart rate [min^–1^]	79.20 [72.73, 85.68]	74.42 [68.33, 80.52]	72.48 [66.69, 78.26]	−4.62, **<0.001**	−0.81 [−1.34, −0.28]	−6.18, **<0.001**	−1.14 [−1.80, −0.48]	79.68 [72.01, 87.35]	73.19 [65.95, 80.43]	74.36 [67.46, 81.25]	−5.43, **<0.001**	−1.10 [−1.77, −0.43]	−4.24, **<0.001**	−0.90 [−1.52, −0.29]
RMSSD [ms]	35.50 [28.23, 42.76]	50.86 [37.55, 64.16]	46.35 [37.92, 54.77]	3.52, **0.002**	1.14 [0.28, 2.01]	3.38, **0.003**	0.81 [0.18, 1.43]	32.39 [23.83, 40.95]	46.65 [31.19, 62.11]	38.55 [28.68 48.43]	2.83, **0.011**	1.06 [0.13, 1.99]	1.67, 0.113	0.46 [−0.16, 1.07]
P2T-RSA	−2.65 [−2.95, −2.36]	−1.61 [−1.88, −1.35]	−2.33 [−2.56, −2.11]	7.70, **<0.001**	2.94 [1.34, 4.54]	2.73, **0.013**	0.92 [0.09, 1.74]	−3.02 [−3.37, −2.68]	−2.40 [−2.71, −2.08]	−2.85 [−3.12, −2.59]	4.03, **<0.001**	1.78 [0.53, 3.03]	1.24, 0.229	0.48 [−0.37, 1.32]

Physiological measures recorded during training were compared with measures of the resting state before (baseline) and 5 min after training (recovery) using robust linear mixed-effect models. The 95% CIs and measurement units, if present, are presented in square brackets. P-values below 0.05 are displayed in bold font. The results show that training stimulated cardiac vagal control in both groups. Effects were greater when biofeedback was used and even persisted after the training. CI, confidence interval; d_m_, effect size calculated by the emmeans R package analogous to Cohen’s d; EMM, estimated marginal mean; RMSSD, root mean square of successive heartbeat interval differences; P2T-RSA, natural logarithm of the respiratory sinus arrhythmia calculated by the peak-to-trough method.

**TABLE 3:** Between-group comparisons of physiological measures.

Measure	Training (*n* = 119)				Recovery (*n* = 119)			
	Difference	z ratio	*p*	Effect size *d*_*m*_	Difference	z ratio	*p*	Effect size *d*_*m*_
Respiration rate [min^–1^]	−4.47 [−5.46, −3.48]	−8.859	**<0.001**	−1.81 [−2.70, −0.92]	−2.87 [−3.86, −1.88]	−5.687	**<0.001**	−1.16 [−1.81, −0.52]
Heart rate [min^–1^]	1.65 [0.15, 3.15]	2.155	**0.031**	0.280 [0.00, 0.56]	−1.46 [−2.96, 0.04]	−1.909	0.056	−0.25 [−0.53, 0.03]
RMSSD [ms]	4.84 [−1.67, 11.35]	1.457	0.145	0.36 [−0.15, 0.87]	7.06 [0.55, 13.57]	2.126	**0.034**	0.53 [−0.01, 1.06]
P2T-RSA	0.71 [0.55, 0.87]	8.913	**<0.001**	2.01 [1.02, 2.99]	0.42 [0.27, 0.58]	5.318	**<0.001**	1.20 [0.51, 1.88]

Comparisons are based on the differences of baseline adjusted estimated marginal means between groups (active control to biofeedback group) derived from robust linear mixed-effect models. The 95% CIs and measurement units, if present, are presented in square brackets. P-values below 0.05 are displayed in bold font. The results indicate that the use of biofeedback had a significant positive effect on respiratory-linked cardiac vagal control and parasympathetic activation during and after training. CI, confidence interval; d_m_, effect size calculated by the emmeans R package analogous to Cohen’s d; RMSSD, root mean square of successive heartbeat interval differences; P2T-RSA, natural logarithm of the respiratory sinus arrhythmia calculated by the peak-to-trough method.

The findings indicate that both groups successfully and to a great extent slowed their breathing and increased levels of RMSSD and P2T-RSA during training. However, only the BG came close to the target value of 6 breaths per minute (0.1 Hz). A training effect was present for some but not for all participants in the CG. For the respiration rate, RMSSD, and P2T-RSA, effect sizes were persistently higher in the BG. In addition, aftereffects (i.e., difference between baseline and recovery) on respiration rate, RMSSD, and P2T-RSA were observed only in the BG. Group comparison of the baseline adjusted training and recovery phases revealed significant large differences in the respiration rate and P2T-RSA for both phases and a moderate difference for RMSSD during recovery (Table 3). Heart rate dropped in both groups below baseline value during training and recovery and was slightly but significantly higher in the BG during training. Inversely, heart rate tended to be lower in the BG during recovery. A *post hoc* mediation analysis of the effects of respiration rate and heart rate on HRV is presented in the Supplementary material C.

### Psychological measures and executive functioning

Average scores and results of the group × test interaction effect derived from the mixed model ANOVAs as well as correlations with P2T-RSA are reported for each score of the psychological scales and executive function test battery in Table 4. Groups did not differ during pretest (*ps* > 0.15) for any measure. Notable group × test interaction effects and correlations with HRV below or close to the significance level were found only for indicators of attention (Error Rate) and short-term memory (Digit Span Forward), but not of processing speed (Processed Target Objects of the revised d2 Test of Attention, Trail Making Test part A, Reaction Time in the Sustained Attention to Response Task), cognitive flexibility (Trail Making part B and B-A), inhibition (Interference Score of the Stroop Color Word Test and Rate of Correct No-Go Response of the Sustained Attention to Response Task), and working memory (Digit Span Backward, Sequencing, Total; Letter Number Sequencing; Working Memory Index Norm Scores). Pre-post comparisons revealed significant and large improvements only in the BG for the Error Rate score, *t*(11) = 2.92, *p* = 0.014, *d* = 0.84, 95% CI [0.27, 2.14], and Digit Span Forward score, *t*(11) = 3.45, *p* = 0.005, *d* = 1.00 [0.48, 1.79]. Linear regression models verified that the pre-to-post-test effect was strongly dependent on the baseline-adjusted P2T-RSA score for the Error Rate, *F*(1,19) = 5.60, *p* = 0.029, $\eta_{\text{p}}^{2}$ = 0.23, and Digit Span Forward score, *F*(1,19) = 8.19, *p* = 0.010, $\eta_{\text{p}}^{2}$ = 0.30.

**TABLE 4:** Psychological scales and executive functions.

Psychological scales and executive functions tests	Biofeedback group		Active control group		ANOVA: Group × test effect			Spearman correlation with P2T-RSA	
				
	Pre-test (*n* = 12)	Post-test (*n* = 12)	Pre-test (*n* = 10)	Post-test (*n* = 10)	*F*(1,20)	*p*	$\eta_{\text{p}}^{2}$	*r*(19)	*p*
**ASQ (0 to 7)**									
Concealing	4.34 (1.39)	4.42 (1.40)	4.46 (2.03)	4.52 (1.71)	0.00	0.978	0.00	−0.24	0.290
Adjusting	4.73 (1.33)	4.64 (1.67)	4.77 (1.72)	4.01 (2.12)	0.93	0.348	0.04	0.06	0.802
Tolerating	4.50 (1.28)	5.30 (0.99)	4.79 (1.45)	5.02 (1.41)	0.62	0.441	0.03	0.23	0.317
**FFMQ (1 to 5)**									
Non-judging	3.23 (0.60)	3.35 (0.92)	3.64 (1.09)	3.62 (0.75)	0.08	0.787	0.00	−0.03	0.887
Non-reacting	3.30 (1.01)	3.60 (0.52)	3.71 (0.67)	3.51 (0.97)	2.12	0.161	0.10	0.12	0.619
Acting with Awareness	3.13 (0.68)	3.64 (0.76)	3.05 (0.74)	3.09 (0.70)	0.92	0.349	0.04	0.37	0.100
Observing	3.35 (0.90)	3.18 (0.95)	3.51 (0.85)	3.86 (0.51)	1.80	0.195	0.08	0.03	0.873
Describing	3.14 (0.90)	3.27 (0.79)	3.53 (0.71)	3.57 (0.75)	0.03	0.864	0.00	−0.01	0.975
**TSCS (21 to 105)**									
Past Total^a^	86.00 (19.50)	89.00 (17.50)	91.50 (2.75)	91.00 (8.25)	0.00	0.971	0.00	0.22	0.347
Present Total^a^	91.00 (8.70)	91.00 (7.25)	91.50 (2.75)	93.00 (7.00)	1.87	0.187	0.09	−0.22	0.334
Future Total^a^	92.50 (7.50)	94.50 (3.25)	96.00 (4.25)	96.00 (6.25)	0.01	0.940	0.00	0.27	0.235
**SART**									
Reaction Time [ms]	345.70 (34.81)	347.72 (35.43)	364.64 (33.18)	375.82 (45.68)	0.29	0.599	0.01	0.19	0.399
Correct No-go Responses [%]	76.24 (12.38)	75.31 (15.82)	73.70 (16.05)	73.33 (13.04)	0.01	0.909	0.00	0.15	0.519
Correct Go Reponses [%]^a^	98.38 (2.08)	98.38 (2.32)	97.92 (8.45)	97.45 (8.10)	0.40	0.537	0.02	0.15	0.512
**Revised d2 Test of Attention**									
PTO Norm Score	**103.42 (12.42)** **	**110.33 (14.37)** **	**100.40 (17.23)** **	**106.60 (16.31)** **	0.08	0.776	0.00	−0.19	0.404
E% Norm Score	**102.33 (12.74)** *	**108.58 (11.80)** *	95.90 (17.31)	93.60 (15.97)	4.16	0.055	0.17	0.52	**0.015**
CP Norm Score	**103.83 (12.39)** **	**113.08 (13.35)** **	**96.70 (11.61)** **	**103.30 (10.76)** **	0.85	0.369	0.04	−0.09	0.971
**Stroop Color and Word Test**									
Interference Score [s]*^b^*	33.35 (15.28)	32.00 (8.38)	42.40 (11.40)	41.75 (11.45)	0.50	0.490	0.02	0.12	0.608
Trail Making Test									
Part B—Part A [s]^a^	25.00 (14.83)	32.00 (19.88)	29.50 (9.45)	32.00 (16.25)	0.23	0.636	0.01	0.33	0.140
Part A [s]	**31.25 (15.05)** *	**21.54 (5.11)** *	**30.95 (11.45)** *	**25.00 (7.32)** *	0.63	0.436	0.03	0.04	0.873
Part B [s]^a^	52.50 (20.70)	52.00 (18.00)	59.95 (5.50)	57.50 (13.75)	0.02	0.884	0.00	0.33	0.145
**WAIS-IV**									
DS Forward Norm Score	**9.67 (2.84)** **	**11.17 (3.01)** **	9.80 (2.39)	9.30 (1.77)	6.10	**0.023**	0.23	0.48	**0.027**
DS Backward Norm Score	11.58 (3.29)	12.42 (2.43)	9.60 (3.24)	10.90 (1.66)	0.19	0.668	0.01	−0.05	0.817
DS Sequencing Norm Score	**11.92 (2.28)** *	**13.25 (2.01)** *	**10.60 (2.27)** **	**11.90 (2.64)** **	0.00	0.954	0.00	−0.07	0.766
DS Total Norm Score	**11.17 (2.79)** *	**12.58 (2.84)** *	10.00 (2.21)	10.70 (1.34)	1.14	0.298	0.05	0.35	0.123
LNS Norm Score	11.75 (3.02)	11.67 (3.73)	10.30 (2.41)	11.00 (3.06)	0.50	0.486	0.03	−0.32	0.152
WMI Norm Score	108.17 (15.11)	112.08 (17.45)	100.80 (12.65)	104.70 (11.53)	0.00	0.997	0.00	−0.06	0.809

Mean scores, the group × test interaction effect, and the correlation with baseline-adjusted level of HRV during training are presented for each behavioral variable. Standard deviations are presented in parenthesis and measurement units, if present, in square brackets. Value ranges in parenthesis indicate the possible numeric outcomes of the corresponding scale. Mean scores in each group that exhibited significant pre-to-post-test differences and exact p-values below 0.05 are displayed in bold font. The results show that episodic memory improvements related to biofeedback-induced parasympathetic stimulation occurred in scores of attention and short-term memory. ANOVA, analysis of variance; P2T-RSA, natural logarithm of the respiratory sinus arrhythmia calculated by the peak-to-trough method; ASQ, Affective Style Questionnaire; $\eta_{\text{p}}^{2}$, partial eta square; FFMQ, Five Facets Mindfulness Questionnaire; TSCS, Tennessee Self Concept Scale; SART, Sustained Attention to Response Test; PTO, Processed Target Objects; E%, Percentage of Errors; CP, Concentration Performance; WAIS-IV, Wechsler Adult Intelligence Scale—Fourth Edition; DS, Digit Span; LNS, Letter-Number-Sequencing; WMI, Working Memory Index.

^a^The median and the inter-quartile-range (in parenthesis) are reported when values were found not to be normally distributed in one of the groups.

^b^The interference score was calculated based on recommendations of Scarpina and Tagini (2017) and included the processing time, corrected, as well as uncorrected errors of all subtests.

**p < 0.01. *p < 0.05.

### Episodic memory responses

#### Encoding phase

Results for measures of the encoding phase (i.e., the number of responses and the response reaction time to stimuli) are presented in the Supplementary material D Table D1. Reaction time values were ln-transformed prior to linear regression due to violations of the normality assumption. No statistically significant effect involving the interaction of group × test was observed for the number of correct responses, self-attributed adjectives, and autobiographical memories recalled. Contrary, the group × test interaction was found to influence the reaction time in each condition. Generally, the BG took more time to respond in the post- than in the pre-assessment, whereas the CG took less time. Consequently, the ln-transformed reaction time was included as a covariate for the analysis of the subsequent memory results.

#### Episodic memory performance

Estimated marginal means and correlations with P2T-RSA of proportions of correct free recalls, subjective R responses, and correct source recollections to studied items (i.e., hits) as well as the discrimination performance between old and new items are listed in Table 5. Groups did not differ significantly for any of the pre-test measures. Moreover, no significant group × test interaction effects, group differences during pre-test measures, or time differences within groups were present for any reference condition for proportions of R responses to unstudied items and incorrect source recollections to studied items (i.e., false alarms). Therefore, R response and source recollection hits were not corrected for false alarms and were treated as indicators of subjective and objective recollection, respectively (c.f., Duarte et al., 2008).

**TABLE 5:** Episodic memory performance.

Memory measures	Biofeedback group		Active control group		ANCOVA: Group × test effect				Spearman correlation with P2T-RSA	
				
	Pre-test (*n* = 12)	Post-test (*n* = 12)	Pre-test (*n* = 10)	Post-test (*n* = 10)	*df*	*F*	*p*	$\eta_{\text{p}}^{2}$	*r*(19)	*p*
**Non-self-referential items**										
Free recall hit rate	0.14 (0.03)	0.18 (0.03)	0.18 (0.03)	0.17 (0.03)	1, 24.2	0.57	0.457	0.02	0.24	0.293
Subjective recollection hit rate	0.41 (0.06)	0.46 (0.06)	0.38 (0.07)	0.48 (0.07)	1, 24.7	0.31	0.584	0.01	−0.03	0.899
Objective recollection hit rate	0.28 (0.04)	0.33 (0.06)	0.13 (0.05)	0.24 (0.06)	1, 24.2	0.43	0.518	0.02	−0.09	0.686
Old/new discrimination (*d*′)	1.07 (0.12)	1.17 (0.15)	1.09 (0.14)	1.16 (0.17)	1, 24.3	0.01	0.935	0.00	0.20	0.377
**Self-referential items**										
Free recall hit rate	**0.16 (0.03)** **	**0.27 (0.03)** **	0.13 (0.03)	0.20 (0.04)	1, 21.4	0.61	0.442	0.03	−0.07	0.776
Subjective recollection hit rate	0.51 (0.08)	0.60 (0.06)	0.53 (0.09)	0.51 (0.07)	1, 21.9	1.43	0.245	0.06	0.33	0.146
Positive items	**0.49 (0.09)** **	**0.71 (0.07)** **	0.53 (0.10)	0.45 (0.08)	1, 22.4	6.90	**0.016**	0.24	0.57	**0.008**
Negative items	0.54 (0.09)	0.50 (0.07)	0.53 (0.10)	0.57 (0.08)	1, 21.0	1.05	0.319	0.05	−0.06	0.789
Objective recollection hit rate	**0.30 (0.07)** **	**0.48 (0.07)** **	0.22 (0.07)	0.24 (0.08)	1, 22.0	3.89	0.061	0.15	0.38	0.092
Positive items	**0.27 (0.08)** ***	**0.52 (0.08)** ***	0.23 (0.08)	0.21 (0.09)	1, 22.6	6.79	**0.016**	0.23	0.43	0.055
Negative items	0.33 (0.07)	0.43 (0.08)	0.21 (0.08)	0.26 (0.08)	1, 21.0	0.05	0.833	0.00	0.22	0.343
Old/new discrimination (*d*′)	**1.50 (0.12)** *	**1.79 (0.11)** *	1.26 (0.14)	1.34 (0.13)	1, 21.4	1.15	0.296	0.05	0.48	**0.031**

EMMs of pre-to-post changes, their correlation (groups combined) with baseline-adjusted level of HRV during training, and the group × test interaction effect are presented for each memory variable. Statistics were derived from robust linear mixed-effect models. The behavior at encoding was controlled for. Standard deviations are presented in parenthesis. 95% CIs are presented in square brackets. EMMs in each group that exhibited significant pre-to-post-test differences and exact p-values below 0.05 are displayed in bold font. Subjective and objective recollection reflect the proportions of correct remember responses and source recollections to studied items, respectively. The results show that episodic memory improvements related to biofeedback-induced parasympathetic stimulation occurred in the recollection of positive items encoded with self-reference. P2T-RSA, natural logarithm of the respiratory sinus arrhythmia calculated by the peak-to-trough method; EMM, estimated marginal means; ANCOVA, analysis of covariance; $\eta_{\text{p}}^{2}$, partial eta square.

***p < 0.001. **p < 0.01. *p < 0.05.

**FIGURE 4:**
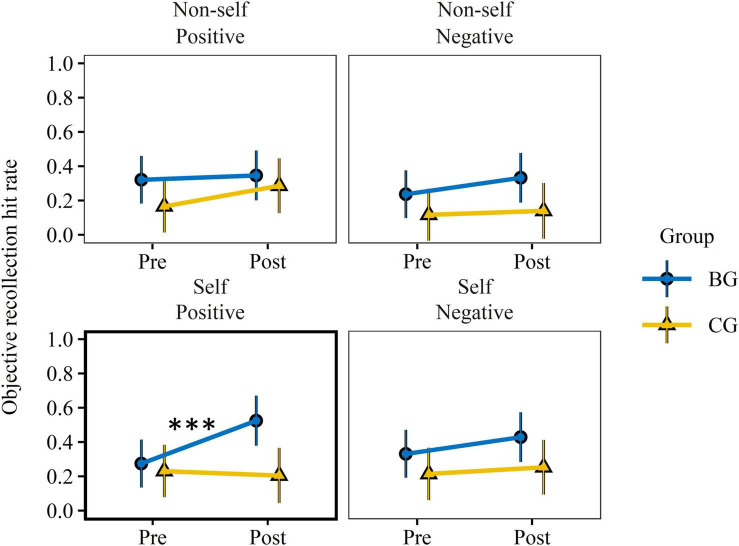
Training effect on objective memory recollection. Objective recollection hit rate was assessed by the proportion of correct source recollection of studied words. EMMs and 95% CIs, derived from robust linear mixed effect models, are presented per group, reference condition, valence, and test. EMMs accounted for inter-participant differences in response reaction time at encoding and random subject effects. Pre-test values did not differ significantly (*p* > 0.14) between groups. Only recollections of positive items encoded with the self-reference condition (bold frame) revealed a significant group × test interaction effect. Training related improvements were found only in the BG. BG, biofeedback group; CG, active control group; EMM, estimated marginal means. ^***^p < 0.001.

A statistically significant group × test × valence effect was present only for the subjective probability estimates of self-referential items, *F*(1, 139.3) = 5.06, *p* = 0.026, $\eta_{\text{p}}^{2}$ = 0.04. None of the measures exhibited a group × test × encoding condition × valence or group × test × encoding condition interaction effect. As subjective and objective recollection are closely related, interaction effects in each level of valence were checked for both variables. Significant group × test interaction effects occurred only in the subjective and objective recollection (see Figure 4) of positive self-referential items. Significant large sized pre-to-post increases were verified only in the BG, *t*(101) = 2.68, *p* = 0.009, *d*_*m*_ = 0.80, 95% CI [0.09, 1.51] (subjective recollection), *t*(138) = 3.40, *p* < 0.001, *d*_*m*_ = 0.98, 95% CI [0.24, 1.72] (objective recollection). Moreover, both scores (groups combined) as well as old/new discrimination of self-referential items correlated significantly or by trend with P2T-RSA. Scores of *d*′ improved only in the BG, *t*(19.9) = 2.26, *p* = 0.035, *d*_*m*_ = 0.61, 95% CI [−0.02, 1.25]. The pre-to-post-test effects were moderately-to-strongly but not statistically significantly dependent on the baseline-adjusted P2T-RSA score for measures of subjective recollection, *F*(1,19.3) = 3.92, *p* = 0.062, $\eta_{p}^{2}$ = 0.17; objective recollection, *F*(1,19.3) = 1.35, *p* = 0.260, $\eta_{p}^{2}$ = 0.07; and *d*′, *F*(1,19.8) = 1.59, *p* = 0.223, $\eta_{p}^{2}$ = 0.07.

To control whether group × time interaction effects were related to changes in other cognitive faculties, previous behavioral measures that exhibited a significant group × test interaction effect (i.e., Error Rate and Digit Span Forward norm scores) were added separately as covariates to the regression model. No effect was observed on any EM variable.

#### Self-reference effect

The follow-up analysis looking at the SRE (i.e., semantic-to-self-episodic differences) revealed no significant group × test but did reveal group × test × valence interaction effects, except for the free recall hit rate, for any EM variable. Group × test interaction effects, EMMs of pre-to-post changes, and their correlation with P2T-RSA are presented for positive and negative stimuli in Table 6. A significant interaction effect and correlation were observable only for subjective and objective recollection of positive stimuli. Pre-to-post comparisons revealed moderate-to-large increases in the BG for subjective recollection, *t*(39.8) = 1.51, *p* = 0.140, *d*_*m*_ = 0.78, 95% CI [−0.29, 1.85], and objective recollection, *t*(46.7) = 2.75, *p* = 0.008, *d*_*m*_ = 1.27, 95% CI [0.18, 2.36], for positive stimuli. Also, a moderate but statistically not significant increase occurred in the SRE based on negative items assessed by *d*′, *t*(74.9) = 1.87, *p* = 0.072, *d*_*m*_ = 0.78, 95% CI [−0.12, 1.68]. The pre-to-post-test effects were strongly dependent on the baseline-adjusted P2T-RSA score for measures of subjective recollection, *F*(1,18.4) = 6.03, *p* = 0.024, $\eta_{p}^{2}$ = 0.25; objective recollection, *F*(1,18.4) = 4.97, *p* = 0.038, $\eta_{p}^{2}$ = 0.21; and *d*′, *F*(1,18.4) = 4.61, *p* = 0.045, $\eta_{p}^{2}$ = 0.20. For both positive and negative items, there was no effect of the Error Rate or Digit Span Forward norm score on the SRE. In comparison, when the SRE was defined as semantic-to-self-semantic differences, the results showed the same trend, but effects were less pronounced (c.f. Supplementary material D Table D2).

**TABLE 6:** Self-reference effect (SRE).

Variable	Pre-to-post change in SRE		ANCOVA: Group × test effect				Spearman correlation with P2T-RSA	
	Biofeedback group (*n* = 12)	Active control group (*n* = 10)	*df*	*F*	*p*	$\eta_{\text{p}}^{2}$	*r*(19)	*p*
**Negative items**								
Free recall hit rate	0.18 (0.10)	−0.03 (0.11)	1, 19.5	2.37	0.140	0.11	−0.04	0.850
Subjective recollection hit rate	−0.07 (0.20)	−0.04 (0.22)	1, 19.6	0.02	0.896	0.00	−0.02	0.942
Objective recollection hit rate	0.04 (0.16)	0.10 (0.17)	1, 19.6	0.08	0.783	0.00	0.18	0.423
Old/new discrimination (*d*′)	0.49 (0.27)	−0.40 (0.29)	1, 19.6	4.90	**0.039**	0.20	0.37	0.094
**Positive items**								
Free recall hit rate	0.08 (0.10)	0.12 (0.11)	1, 19.2	0.22	0.642	0.01	−0.27	0.236
Subjective recollection hit rate	0.30 (0.20)	−0.37 (0.22)	1, 19.4	6.88	**0.017**	0.26	0.45	**0.038**
Objective recollection hit rate	0.43 (0.16)**	−0.25 (0.17)	1, 19.4	8.49	**0.009**	0.30	0.47	**0.031**
Old/new discrimination (*d*′)	0.31 (0.27)	0.51 (0.29)	1, 19.4	0.24	0.627	0.01	0.09	0.696

EMMs and group differences of pre-to-post changes in the SRE. The SRE is defined here for each variable as the episodic memory score difference from the semantic to the self-episodic condition. EMMs of pre-to-post changes, their correlation (groups combined) with baseline-adjusted level of HRV during training, and the group × test interaction effect are presented for each memory variable. Statistics were derived from robust linear mixed-effect models. The behavior at encoding was controlled for. Standard deviations are presented in parenthesis. 95% CIs are presented in square brackets. EMMs in each group that exhibited significant pre-to-post-test differences and p-values below 0.05 are displayed in bold font. Subjective and objective recollection reflect the proportions of correct remember responses and source recollections to studied items, respectively. The results showed that increases in the SRE related to biofeedback-induced parasympathetic stimulation occurred in the recollection of positive items, confirming previous results. EMM, estimated marginal means; ANCOVA, analysis of covariance; $\eta_{\text{p}}^{2}$, partial eta square.

**p < 0.01.

### Exploratory analysis

Previous psychophysiological research has linked cognitive processes typically to resting-state measures of vagally mediated HRV. In the framework of an unplanned analysis, the monotonic relationship between vagally mediated resting-state HRV indexed by lnHF with behavioral results was investigated. Scores of lnHF and respiration rate that affect to which degree vagal influences are captured by lnHF, did not exhibit any group, session, or group × session effects. Therefore, physiological baseline indicators were grouped by participants and EMMs were correlated with the pre-test scores using the Spearman and two-sided hypothesis tests. None of the psychological, executive functions, EM or SRE measures correlated with lnHF with the exception of the Stroop global interference score, *r*(19) = −0.49, *p* = 0.025; Describing score of the Five Facets of Mindfulness Questionnaire, *r*(19) = −0.44, *p* = 0.025, and the correct free recall rate of positive self-referential items, *r*(19) = 0.67, *p* = 0.001.

## Discussion

The objective of this study was to determine the placebo-controlled and 1-week lasting effect of HRVB on executive functions and self-referential EM in young healthy adults. Beyond that, it was investigated whether changes in cognitive performance were linked to RSA amplitude during training as a measure of respiratory-related CVC and training success. The results show that biofeedback had a large effect on RSA amplitude during and after training and some cognitive functions. Group × test interaction effects were found for measures of attention, short-term memory, and self-referential EM of positive stimuli. VR-related properties were controlled for. Moreover, findings indicated that RSA amplitude mediated cognitive improvements, thus confirming our hypotheses. However, some scores of affective and executive control were not affected by HRVB, including working memory and inhibitory control. Neural structures that underlie the implication of HRVB on self-reference processing are discussed.

### Physiological outcomes

Physiological outcomes were in line with the Psychophysiological Coherence (McCraty and Childre, 2010) and Resonance Frequency Training (Lehrer et al., 2000) models. The training program proved effective for both groups, with a clear difference in training effect. The results showed, as hypothesized, that HRV self-regulation training evoked greater RSA amplitude during and even after the training when biofeedback was provided. Consistent with our assumption, the biofeedback effect was higher on RSA amplitude than RMSSD during training. In fact, biofeedback did not influence RMSSD during training, only afterward. These findings indicate that the biofeedback effect on CVC during training was strongly related to changes in respiration eliciting physiological coupling between respiratory, blood pressure, and cardiac phases. *Post hoc* analyses confirmed that slower respiration, along with lower heart rate, mediated to the greatest part the training and aftereffects on RSA amplitude. On the other hand, RMSSD was influenced by heart rate but not by respiration rate (Supplementary material C). While physiological coupling may be assumed during HRVB due to breathing near the resonance frequency, it remains unclear whether aftereffects on respiration rate and RSA amplitude were also linked due to increases in coherence. Besides, stronger ventilation (i.e., tidal volume), which is typical for HRVB, might have affected RSA amplitude but RMSSD less so. The observation that no significant group difference was demonstrated for RMSSD during training may be inferred from three assumptions. Firstly, as demonstrated by the mediation analysis, RMSSD is relatively unaffected by changes in respiration. Secondly, RMSSD was biased due to *cycle length dependence* (i.e., dependence of HRV on the heart rate; McCraty and Shaffer, 2015): higher heart rate reduced RMSSD in the BG. Thirdly, biofeedback evoked greater attention-driven sympathetic activation. With respect to the latter two points, we assume that biofeedback triggered greater motivation toward the engagement in voluntary control of the respiration during training. Evidence supporting this claim was provided by group differences in breath control and self-reported attentional commitment to the task that were higher in the BG. In turn, directing attention to a task is linked to increased arousal and sympathetic activation which decreases RMSSD and increases heart rate. We suppose that, mediated by attention, voluntary breath control, and tidal volume, biofeedback stimulated sympatho-vagal efferent cardiac inputs for both branches of the nervous system. In this line of thought, sympathetic activation during training partly counterbalanced the vagally mediated influence on RMSSD. This assumption is supported by the finding that RMSSD values between groups did differ after training. This suggests that in the BG the completion of the task was accompanied by sympathetic deactivation and thus a greater parasympathetic shift in the sympatho-vagal balance. The same pattern was reflected by the mean heart rate which reflects mean efferent vagal and sympathetic effects. Heart rate was higher during training in the BG, despite greater parasympathetic-mediated HRV (RSA amplitude, SDNN), and continued to drop after training only in the BG.

Conclusively, the outcomes demonstrate for the first time the moderating effect of biofeedback signals on the autonomic changes elicited by HRV self-regulation during and beyond the moment of self-regulation training. We therefore assume that biofeedback supported parasympathetic activation through increases in respiratory-linked CVC.

### Executive functioning

In accordance with previous HRVB studies, the outcomes indicate that HRVB improves attentional capabilities required for the identification and discrimination of external stimuli and auditory short-term memory but not cognitive flexibility (Pop-Jordanova and Chakalaroska, 2008; Jester et al., 2019) and working memory (Lloyd et al., 2010) in young healthy adults. Moreover, these cognitive changes could be linked to ANS processes during training. This suggests that neurophysiological stimulation and not any placebo effect triggered cognitive enhancement. This idea is in line with previous findings that demonstrated a link between vagally mediated HRV and selective attention (Giuliano et al., 2018) as well as with the discrimination performance of false from true memories (Feeling et al., 2021). A change of response strategy that may change the interpretation of these results seems unlikely as speed and accuracy with respect to go-/no-go responses during the Sustained Attention to Response Task did not change from pre- to post-test in either group. Moreover, EM results support the idea that parasympathetic stimulation improved discrimination performance, not only of external stimuli, but also of internal thought processes as HRV significantly correlated with old/new memory discrimination performance. Future studies should check other types of working memory such as visuospatial span.

Notable consideration has been given to the association of resting-state vagally mediated HRV and HRVB with inhibition processes on neurophysiological and behavioral levels. In accordance with previous findings (Gillie et al., 2014; Feeling et al., 2015; Bernardi et al., 2016), results of the exploratory analysis showed a positive link between vagally mediated resting-state HRV and inhibitory control. Consequently, one would expect that, as for the identification and discrimination performance, stimulating physiological coherence and CVC would lead to greater inhibition. Yet, contrary to previous literature (Sherlin et al., 2010; Sutarto et al., 2010, 2013; Prinsloo et al., 2011; Blum et al., 2019), no training effects on inhibitory control were observed in either group, irrespective of whether the task required motor components (Sustained Attention to Response Task) or not (Stroop Color and Word Test). However, as in this investigation, studies controlling for the biofeedback training effect failed to report any significant interaction effects or group differences (Sherlin et al., 2010; Sutarto et al., 2010, 2013; Prinsloo et al., 2011). Hence, the lasting benefits of HRVB on behavioral inhibitory control mechanisms remain unsubstantiated. One probable criterion might be insufficient sample size, which ranged from 9 to 60 in previous studies, or training intensity.

### Episodic memory and self-reference

Memory performance outcomes of the self-reference episodic memory task suggest that vagally mediated effects of HRVB can improve recognition-based verbal EM through the strengthening of the SRE. On the one hand, placebo-controlled training benefits in the BG were present only when recollecting self-referentially encoded positive items. On the other hand, memory recollection and discrimination scores correlated moderately-to-strongly with HRV. In addition, advantages in memory retention of self-referential stimuli did not come at the cost of reduced memory performance of non-self-referential stimuli. Another indicator of the involvement of the self-processing system is the discrepancies between memory measures. Of all memory variables here, recollection success relies the most on self-referential memory because it requires the episodic recollection of information associated with the stimulus during encoding. To this effect, Conway and Dewhurst (1995) and Conway et al. (2001) even introduced the term self-reference recollection effect (SRRE) that comprises the SRE on the subjective and objective recollection measures. In addition, there are differences between the two recollection measures. True recollection can be assured for the measure of objective recollection (i.e., source retrieval) but not always for subjective recollection. Subjective remembering is often confused with high-confidence recognition, which does not necessarily rely on the recollection of qualitative properties of the memory (Yonelinas, 2001; Rotello et al., 2005). Discrepancies in the biofeedback effect (i.e., group × test interaction effect) and correlation results between memory measures of self-referential items might therefore stem from different degrees of dependence on self-reference processes.

The importance of the self-processing system for the memorial training effect is further stressed by the findings related to the SRE. On the one hand, the presence of biofeedback had a large promoting effect on the SRRE (SRE on subjective or objective recollection) on both scales, providing evidence for the interaction between biofeedback and self-reference processing. On the other hand, results of the SRE reflected the same characteristics as those of the memory results discussed above: firstly, changes in the SRRE were linked to HRV; secondly, differences occurred between memory variables with effect sizes and correlations that were markedly larger for the recollection measures.

**FIGURE 5:**
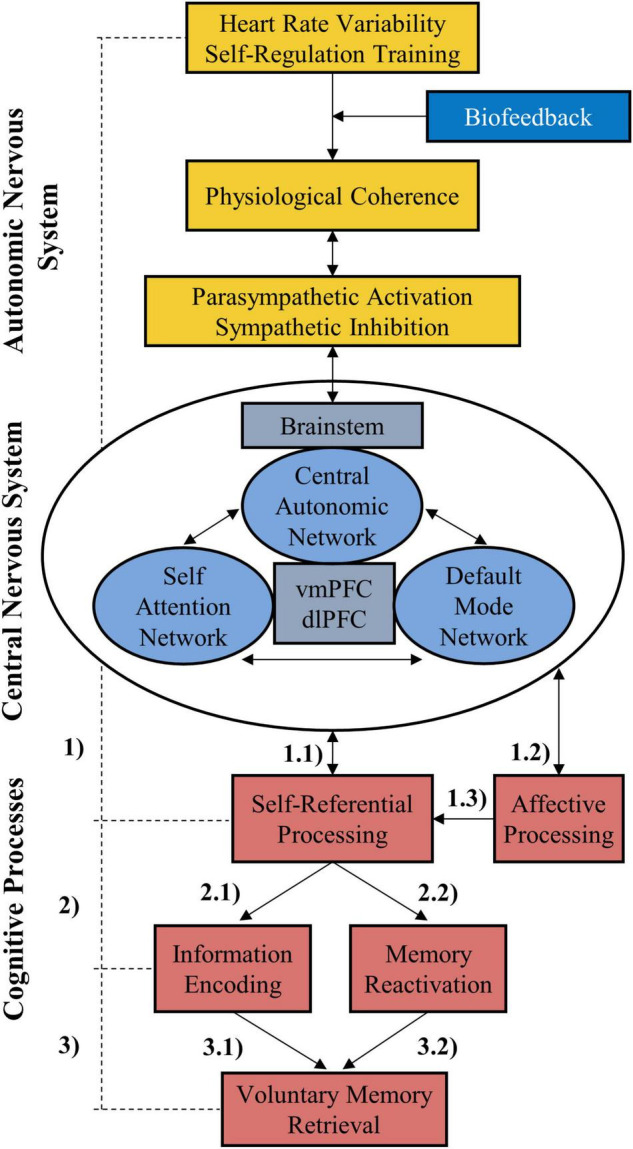
The self-regulation-self-referential-memory (SR-SRM) model. Autonomic control can be exercised through HRV self-regulation training and thus affects pathways of the autonomic nervous system which reach the central nervous system. These pathways connect *via* the brainstem with several forebrain structures in the central autonomic network. Due to shared structures (most notably the vmPFC) further brain networks are influenced down the line which are involved in self-referential information processing (1.1). Likewise, affective processing is affected by functional changes related to limbic structures (1.2) which in turn influence self-referential processing (1.3). Firstly, self-referential encoding (2.1) provides a memory advantage to the encoded information (3.1). Secondly, self-referential processes are associated with the internal focus on thoughts that can trigger reactivation of a new memory or add novel associations to it, especially when the memory is related to the self (2.2), supporting its consolidation and retrieval (3.2). Consequently, strengthening self-referential processing *via* HRV self-regulation training may support voluntary retrieval of memory. In this context, biofeedback acts as a moderator because it facilitates self-regulation. vmPFC, ventromedial prefrontal cortex; HRV, heart rate variability.

Remarkably, training effects on the SRRE concerned only positive items. This is an indication that affective biases played a role in the influence of biofeedback and HRV self-regulation training on SRRE. Indeed, it is a well-documented phenomenon that self-relevant stimuli produce a bias in positivity, such that people tend to make flattering self-evaluations and to hold more positive memories (see Cunningham and Turk, 2017, for a review on self-referencing biases). Furthermore, memory improvements were not mediated by changes in any other cognitive function assessed in this study. These results suggest that vagal stimulation and increased physiological coherence also mediated an effect of HRVB on cognitive processes beyond executive functioning associated with improved objective memory recollection and discrimination performance. Moreover, it supports the notion of a direct relationship between autonomic control and the self-reference processing system that may be based on a common set of neural structures. We propose a chain mediation model explaining the effect of HRV self-regulation training on the memory retrieval of self-referential encoded information (Figure 5).

### Self-referential episodic memory and autonomic control

Within the central nervous system, there are several brain structures that are commonly engaged in autonomic control, self-reference, and emotional processing such as the vmPFC, dorsolateral prefrontal cortex (dlPFC), or cingulate cortex. The former is particularly noteworthy due to its central role in brain networks orchestrating autonomic and self-reference processes. On the one hand, the vmPFC as well as several other prefrontal regions including the dlPFC, cingulate cortex, and left anterior insula function as nodes of the central autonomic network (Benarroch, 1993; Sklerov et al., 2019) at the highest level of a top-down chain to control autonomic functions such as cardiac regulation. On the other hand, the vmPFC, as part of the Self-Attention Network (SAN; Humphreys and Sui, 2016), has been firmly linked to self-reference processes (Kelley et al., 2002; Macrae et al., 2004; Northoff et al., 2006; Martinelli et al., 2013). The SAN is believed to be responsible for self-biases in perception and attention through the activation of the vmPFC which is moderated by a control network involving the dlPFC. Moreover, the dual role for self-reference and autonomic processes is further underlined by the functioning of the default mode network (DMN; Uddin et al., 2009). The DMN is typically associated with self-referential cognition (Buckner et al., 2008) as it is active at moments of rest when attention is not directed to the exterior environment but to the self. Besides, the DMN is constituted mainly of brain regions involved in autonomic processing, with the vmPFC as one of its core centers, and is in direct relationship with autonomic control (Beissner et al., 2013; Bär et al., 2015).

Consequently, within the autonomic nervous system, it seems possible that cardiac regulation during HRVB training influenced the functional dynamic of the vmPFC and related pathways. Indeed, the vmPFC is linked to antisympathetic and parasympathetic ANS activity, whose elicitation was the aim of the training. In this context, biofeedback acts as a moderator that supports physiological change. The existence of a reciprocal interaction between the autonomic nervous system and forebrain in the context of cognition, memory, and emotion that strongly relies on central autonomic network elements has also been emphasized earlier (Thayer and Lane, 2000, 2009; Thayer et al., 2009; Critchley et al., 2013). Direct evidence that HRVB training increases resting state functional connectivity in the forebrain and between regions of the central autonomic network was recently demonstrated by Schumann et al. (2021). They showed that HRVB intervention compared to a control group increased connectivity between vmPFC and several forebrain structures, including the cingulate cortex, left anterior insula and dlPFC. They also found that the effect was mediated by elements of the central autonomic network in the brainstem. Hence, it can be assumed that repeated HRVB training may have a sustained impact on the dynamism of the self-reference system due to surges in functional connectivity between forebrain structures (i.e., vmPFC, dlPFC, etc.) and activity in the vmPFC which in turn can be ascribed to the promotion and inhibition of pathways involved in parasympathetic and sympathetic autonomic regulation, respectively.

In this context, we theorize that these functional changes (see Figure 5 “CNS”) evoked by HRV self-regulation training (see Figure 5 “ANS”) facilitated the processing of self-referential information at (1.1) or after initial encoding (2), which leads to the inflation of the SRE and self-related memory retrieval (3). According to the neurophysiological assumptions postulated here, there is also an alteration in the functionality between the vmPFC and limbic structures involved in affective processing and reward circuitry (e.g., cingulate cortex, insula, and amygdala). Moreover, vmPFC activity has been linked to affective meaning and positive valence ratings (Winecoff et al., 2013; Kim and Johnson, 2015). Hence, we postulate that functional changes were also responsible for changes in affective processing (1.2), explaining the positivity bias in the presented results. This is in line with previous literature that repeatedly linked HRVB to changes in emotional processing (McCraty et al., 2009; Lehrer et al., 2020). According to the Psychophysiological Coherence model (McCraty and Childre, 2010), activating positive emotions harmonizes bodily processes that shift an “inner baseline reference” (McCraty and Zayas, 2014) related to alterations in information processing. So, following the HRVB training, which likewise stimulated physiological coherence, may have also resulted in such a baseline shift, biasing information processing toward positive stimuli. In this regard, HRV self-regulation training influences affective arousal elicited by positive stimuli when PFC is engaged in self-reference processes impacting subsequent information processing (1.3).

Previous literature suggests that the SRE is primarily related to processes at encoding (Morel et al., 2014; Kalenzaga et al., 2015) (2.1 and 3.1). Moreover, following our assumptions, biofeedback can also be surmised to affect DMN functionality, impacting information processing related to our default mode. Besides the inherited central aspect of self-reference, the default mode is also concerned with a range of self-relevant cognitive processes such as mind wandering, recalling memories, simulating, or planning the future (Mooneyham and Schooler, 2013). Hence, the training may also have important consequences on the behavior related to the default mode. For instance, subjects might be more prone to engage in self-referential cognition or to be more concerned by affective biases. In this regard, an alternative interpretation of our memory results is that training affected self-referential thought processes occurring after the initial encoding during the consolidation or retrieval phase (2.2). It might be that the training success was associated with a greater propensity to engage in retrospection or prospection whose content was linked to the learned self-referential memory probes triggering memory reactivation and reassociation. Consequently, these processes could have facilitated access to the memory trace at retrieval (3.2). Previous findings underline that post-encoding memory consolidation can occur at rest (Axmacher et al., 2008; Tambini and Davachi, 2013) and when engaged in a task with high frontal related processing demands but low hippocampal related processing demands (Varma et al., 2017, 2018). One indicator to verify whether HRVB promoted self-reference processes occurring after encoding, and thus after post-encoding memory consolidation, may be the comparison of pre-to-post changes in the SRE between the recollection measures and free recall success for positive items. Based on the assumption that an essential part of the memory consolidation took place during the long retention period between the free recall and recognition phases, one would expect in the BG a higher pre-to-post-increase in the SRE for the measure of objective recollection than for free recall success, which was the case in this work. It is thus likely that improvements in EM retrieval were mediated by altered processes at both stages, during encoding and after encoding. Though the influence of encoding processes on the SRE is scientifically more founded, it remains unclear to what extent HRVB related memory improvements can be attributed to these two stages, respectively.

### Covariates

In the current study the influence of HRVB on various measures of psychological trait characteristics including emotional regulation, mindfulness, or self-concept was checked. Neither the HRVB training nor the mindfulness training in the CG showed an influence on any score. Accordingly, contrary to previous assumptions, no biofeedback-related changes in affective control or self-awareness were verified. One likely explanation is that the scales used were not sensitive enough to capture any changes as they assessed stable traits rather than reactional states. Furthermore, it is possible that the intervention period and/or the intensity was not long enough or high enough to produce trait changes that could be perceived by the participant.

Experimental biases exhibited by behavioral changes at encoding in terms of response reaction time and number of adjectives associated with the self-concept or autobiographical memories can be excluded since measures were added as covariates to the statistical models when necessary. Furthermore, one might argue that the training affected interoceptive awareness that mediated the impact of the bodily state on memory. In other words, participants were more conscious of autonomic processes causing additional information to be linked to memory. Therefore, additional qualitative properties or cues that had been deeply encoded *via* integration with autonomic control (Critchley et al., 2013) may have been available, facilitating retrieval. The moderate correlation found between HRV and the measure of awareness supports this claim. Also, Blum et al. (2019) found that a VR-immersion with a natural scene during HRVB increases aspects of a mindfulness state. However, given the statistical insignificance of the correlation and the fact that no effects were observed for any of the mindfulness trait scales, the outcomes did not confirm that self-awareness was related to memory encoding. Nevertheless, it cannot be ruled out that measures of mindfulness state or interoceptive awareness instead of general trait features might have accounted for variations within the results.

In the present study it has been shown that the VR setup used evoked comparable levels of sense of embodiment and presence between groups. We therefore suspect that these characteristics did not moderate group differences in instantaneous and potential long-term psychophysiological changes. Especially self-ownership, which can also extend to the avatar and embodied biofeedback, is known to affect the SRE through the activation of brain networks involved in self-processing (Cunningham et al., 2011; Cunningham and Turk, 2017). However, we do not exclude the possibility that the use of VR moderated HRVB effects through other VR-related mechanisms. For instance, it is possible that the VR implementation increased bodily self-consciousness (Blanke, 2012) during HRVB, and therefore supported self-processing, through greater attention to the biofeedback signal. Though the direct effect of the VR on HRVB outcomes was not assessed in this study, the large effect sizes indicate that the use of VR enhanced cognitive improvements when compared to standard-HRVB, especially regarding the SRE. This effect might be corroborated by more frequent training and will be the subject of future studies which contrast VR-HRVB with a classical HRVB intervention.

Another important factor for the mediation of assessment score changes was participants’ expectations about the training efficacy. Subject-expectancy effects are known to contribute to HRVB outcomes (Khazan, 2013) and were therefore controlled through blinding and introduction of an active placebo-control group. Furthermore, it has been argued that subject-expectancy effects may be influenced by the perception of control over physiological parameters (Weerdmeester et al., 2020). The finding that groups did not differ in terms of agency, however, is suggestive that participants receiving biofeedback did not expect their training to be more beneficial due to a stronger perception of any changes that their self-regulation training evoked. In addition, results of the online follow-up questionnaire (Supplementary material E) hinted that expectations about cognitive training effects did not differ between groups.

### Implications and perspectives

One of the strengths of the current work is that contrary to most HRVB studies, post-evaluations were not carried out immediately after the last training session. It is therefore probable that certain training effects were not observable due to their ephemerality. This might also explain why here no effect was reported on several executive functions, specifically inhibitory control, whereas it was reported in studies with comparable sample size and that did not include a maintenance period. Additionally, in contrast to other studies, a statistical approach was adopted that is robust against group differences and violations of distributional assumptions, that accommodates for random subject and time effects (Schielzeth et al., 2020), providing reliable Type 1 error rates for small samples. Consequently, the findings emphasize that instant effects cannot be simply extrapolated, and that caution should be exercised when interpreting such results. Further, this underlines the importance of including a maintenance period in experimental paradigms that investigate the effect of biofeedback on cognition.

Moreover, previous HRVB studies investigated mainly (young) adults who were susceptible to or experienced high levels of work-related stress. Those studies postulated that through the reduction of stress, cognitive performance increases. The present work shows that improvements can also be evoked in young adults without any specified pre-condition and that no experience in self-regulation training is required. The findings also demonstrate for the first time that only six 25-min sessions of HRVB training can be sufficient to provoke changes in the self-referential EM system and discrimination performance that are sustained beyond the training period. Thus, the present study stresses the importance of VR-HRVB interventions in yielding potential long-term advantages for cognition and well-being, especially in regard to short- and long-term memory and committing errors. However, these results have yet to be corroborated by protocols including longer training and maintenance periods, samples of different age segments, and clinical populations. Moreover, the influence of VR-related effects needs to be further investigated.

Heart rate variability biofeedback appears as a promising application for a wide range of fields that are concerned with the peak performance, maintenance, or recovery of cognitive functions. For example, HRVB may proof useful as a therapeutic tool to treat patients suffering from impaired cognition or to combat neurocognitive decline. Specifically, those with conditions that distort self-reference and affective processing, such as schizophrenia, may benefit from HRVB. Further, we recommend considering the implementation of biofeedback also for other self-regulation training programs as state-awareness proved to be advantageous for training outcomes.

In addition, if HRVB indeed has a long-lasting effect on the self-reference system and DMN functionality, training programs could have meaningful implications on a wide range of behaviors, such as those related to self-regulation, body self, mind wandering, prospection-based cognition, and social behavior. Besides the demonstrated leads, further investigation is required to ascertain the relationship of changes in HRV with self-referential cognition, short-term memory and the identification and discrimination of stimuli. It would also be interesting to explore whether cognitive processes associated to HRVB can impact memory at later memory phases (i.e., beyond encoding). We encourage future HRVB studies to include resting state or phasic HRV measures shortly before or during cognitive assessment that could explain training related effects. Moreover, multimodal neuroimaging approaches are needed to comprehensively explore the neural dynamism underlying the relationship between HRVB-stimulated autonomic activity and cognition.

## Conclusion

In the framework of this study, a new cognitive training system was developed that allows for human immersion in a naturalistic VR environment in which HRV and respiratory biofeedback can be visualized. For the first time, two randomized and blind groups were compared that trained HRV self-regulation either with or without VR-HRVB. Statistically significant cognitive effects that persisted 1 week after participation in an HRV self-regulation training program were assessed. Findings demonstrate for the first time the essential role of HRVB to drive cognitive improvement through physiological change. Biofeedback improved aspects of attention, short-term memory, and self-referential EM but not inhibitory control, processing speed, working memory and non-self-referential EM. Furthermore, biofeedback greatly strengthened the SRE for positive stimuli. Cognitive improvements could be linked to the level of the respiratory sinus arrhythmia during training which is associated with physiological coherence, baroreflex gain, and CVC. The presented results are in accordance with the neurovisceral integration model providing additional evidence that parasympathetic activation improves cognitive functioning involving the identification and discrimination of stimuli and self-referential processing. It is conjectured that changes in neural pathways and structures related to autonomic control affected brain networks involved in attentional control, affective processing, and self-referential processing. The results provide reasons to direct further research toward the influence of HRVB on the mind-body complex, as it could reveal important implications for neurocognitive functioning and behavior.

## Data availability statement

The datasets presented in this study and details about the virtual reality biofeedback training can be found in the Open Science Framework (osf.io) online repository (doi: 10.17605/OSF.IO/U4T7P).

## Ethics statement

Ethical review and approval was not required for the study on human participants in accordance with the local legislation and institutional requirements. The participants provided their written informed consent to participate in this study.

## Author contributions

LB contributed to conceptualization, methodology, software, validation, formal analysis, investigation, resources, data curation, writing—original draft preparation, writing—reviewing and editing, visualization, and project administration. IC-B contributed to investigation and writing—reviewing and editing. PP contributed to conceptualization, methodology, resources, writing—reviewing and editing, supervision, and funding acquisition. All authors contributed to the article and approved the submitted version.

## Abbreviations

HRVB: heart rate variability biofeedback
ANS: autonomous nervous system
HRV: heart rate variability
CVC: cardiac vagal control
RSA: respiratory sinus arrhythmia
VR: virtual reality
SRE: self-reference effect
EM: episodic memory
vmPFC: ventromedial prefrontal cortex
dlPFC: dorsolateral prefrontal cortex
BG: biofeedback group
CG: active control group
BMI: new body mass index
ECG: electrocardiogram
R/K/G: Remember/Know/Guess
NN: normal-to-normal: time interval between successive normal heartbeats
RMSSD: root mean square of successive heartbeat interval differences
SDNN: standard deviation of normal heartbeat intervals
pNN50: percentage of successive normal heartbeats that differ by more than 50 ms
lnLF: natural logarithm of absolute low frequency power of the heart rate signal
lnHF: natural logarithm of absolute high frequency power of the heart rate signal
normLF: relative power of the low frequency band
normHF: relative power of the high frequency band
LF/HF: absolute power ratio of the low frequency and high frequency band
P2T-RSA: natural logarithm of the RSA calculated by the peak-to-trough method
PB-RSA: natural logarithm of the RSA calculated by the Porges-Bohrer method
ANOVA: analysis of variance
EMM: estimated marginal mean
DV: dependent variable.
