# Supplementary material for: Respiratory sinus arrhythmia during biofeedback is linked to persistent improvements in attention, short-term memory, and positive self-referential episodic memory
Source: Front Neurosci. 2022 Sep 13;16:791498. doi: 10.3389/fnins.2022.791498 (PMC9514056; doi:10.3389/fnins.2022.791498)
Supplement: Supplementary file 1 [file Data_Sheet_1.pdf]

frontiers

Supplementary Material A

Virtual Reality Heart Rate Variability Biofeedback System Assessment

Group mean values and group comparisons of subjective ratings of embodiment, presence, attention/drowsiness, and fatigue are presented in Table A1. Except for the fatigue item, which was presented twice to the participant (before and after VR-immersion), all scales were completed directly after training and in the following order: fatigue item, attention item, drowsiness item, presence questionnaire, embodiment questionnaire. Items of the Slater-Usoh-Steed questionnaire (Slater & Steed, 2000; Usoh et al., 2000) and embodiment questionnaire (Gonzalez-Franco & Peck, 2018) were slightly adapted for the purpose of this work and can be viewed along with the attention and drowsiness items in the materials section of the public repository (Bögge et al., 2021).

Table A1 – Group Results of the System Assessment

Note. Standard deviations are presented in parenthesis. Possible score ranges are indicated for each scale. Mean scores were compared between groups. BG = biofeedback group; CG = active control group; d_m = effect size calculated by the *emmeans* R package analogous to Cohen's d, CI = confidence interval.
[a] The BG were asked additional questions following the recommendation of Gonzalez-Franco and Peck (2018) that addressed their sense of embodiment in terms of response to the biofeedback in the virtual reality environment. Averaged embodiment scores including and excluding the response subscale were calculated.
$^{**}p < .01$. $^{*}p < .05$.

Virtual reality scales	Mean		Group comparison (CG to BG)				
	BG (n = 27)	CG (n = 20)	*df*	*t*	*p*	d_m	d_m 95% CI
Embodiment (-3 to 3)							
Ownership	0.88 (1.02)	0.78 (1.15)	13.9	0.44	.666	0.12	[-1.27, 1.66]
Agency	-0.01 (1.31)	-0.69 (0.85)	14.1	1.11	.287	1.03	[-0.87, 2.93]
Location	1.46 (0.93)	1.38 (0.93)	13.2	0.50	.626	0.11	[-0.67, 0.88]
Appearance	0.15 (1.44)	-0.13 (1.35)	13.9	0.39	.704	0.30	[-1.13, 1.73]
Response [a]	1.07 (1.18)	-	-	-	-	-	
Total (excluding response)	0.69 (0.68)	0.40 (0.61)	14.1	0.97	.348	0.82	[-1.04, 2.69]
Total (including response)	0.73 (0.65)	0.40 (0.61)	14.3	1.12	.283	1.04	[-0.97, 3.04]
Presence (1 to 7)	3.96 (1.53)	4.06 (1.36)	14.1	0.24	.814	-0.19	[-1.92, 1.54]
Attention (1 to 7)	5.72 (0.19)	4.61 (0.21)	14.0	3.89	**.002****	1.01	[0.27, 1.76]
Drowsiness (1 to 7)	2.22 (0.23)	3.39 (0.26)	14.1	-3.41	**.004****	-0.53	[-1.08, 0.02]
Fatigue (0 to 10)							
Before training	2.26 (1.89)	3.22 (2.31)	14.0	-1.24	0.235	-0.57	[-1.58, 0.45]
After training	2.67 (2.25)	4.20 (2.14)	14.2	-2.39	**0.031***	-0.73	[-1.54, 0.01]
Before-to-after comparison	$t(109) = 1.36$<br>$p = .178$<br>$d_m = 0.26$	$t(109) = 2.83$<br>$p =$ **.006****<br>$d_m = 0.63$					

References

Bögge, L., Colás-Blanco, I., & Piolino, P. (2021). BIOTRAC-1 Study: Investigating the persistent effect of heart rate variability biofeedback and changes in vagal tone on executive functions and self-referential episodic memory. *Open Science Framework*. https://www.doi.org/10.17605/OSF.IO/U4T7P

Gonzalez-Franco, M., & Peck, T. C. (2018). Avatar Embodiment. Towards a Standardized Questionnaire. *Frontiers in Robotics and AI*, *5*. https://doi.org/10.3389/frobt.2018.00074

Slater, M., & Steed, A. (2000). A Virtual Presence Counter. *Presence: Teleoperators and Virtual Environments*, *9*(5), 413–434. https://doi.org/10.1162/105474600566925

Usoh, M., Catena, E., Arman, S., & Slater, M. (2000). Using Presence Questionnaires in Reality. *Presence: Teleoperators and Virtual Environments*, *9*(5), 497–503. https://doi.org/10.1162/105474600566989
