# Supplementary material for: Respiratory sinus arrhythmia during biofeedback is linked to persistent improvements in attention, short-term memory, and positive self-referential episodic memory
Source: Front Neurosci. 2022 Sep 13;16:791498. doi: 10.3389/fnins.2022.791498 (PMC9514056; doi:10.3389/fnins.2022.791498)
Supplement: Supplementary file 2 [file Data_Sheet_2.pdf]

Supplementary Material B

Additional Measures of HRV

In accordance with guidelines for heart rate variability (HRV) measurements in psychophysiological research (Laborde et al., 2017), additional indicators of HRV are reported.

Table B1 – Within-Group Comparisons of Additional HRV Measures

Note. Physiological measures recorded during training were compared with measures of the resting state before (baseline) and 5 min after training (recovery). The 95% CIs are presented in square brackets. P values below .05 are displayed in bold font. The additional HRV indicators confirmed that biofeedback training strongly elicited cardiac regulation through respiration-driven activation of the parasympathetic branch during and after training. As expected, significant changes in the high frequency power band only occurred in the active control group as this band reflects parasympathetic activation only when the breathing rate is above 8.5 cycles per minute. Smaller but still significant vagally-mediated effects were also identified in the controls. HRV = heart rate variability; CI = confidence interval; EMM = estimated marginal mean; pNN50 = percentage of successive normal heartbeat time intervals that differ by more than 50 ms; SDNN = standard deviation of successive normal heartbeat time intervals; lnLF = natural logarithm of absolute low frequency power of the heart rate signal; lnHF = natural logarithm of absolute high frequency power of the heart rate signal; LF-norm = relative power of the low frequency band of the heart rate signal; LF-norm = relative power of the high frequency band of the heart rate signal; LF/HF = absolute power ratio of the low and high frequency bands of the heart rate signal, PB-RSA = natural logarithm of the respiratory sinus arrhythmia calculated by the Porges-Bohrer method.
$^{***}p < .001$. $^{**}p < .01$. $^{*}p < .05$.

HRV measure	Biofeedback group							Active control group						
	Baseline (n = 68)	Training (n = 68)	Recovery (n = 68)	Training-Baseline		Recovery-Baseline		Baseline (n = 51)	Training (n = 51)	Recovery (n = 51)	Training-Baseline		Recovery-Baseline	
	EMM	*EMM*	*EMM*	t(19.0), p	Effect size d_m	t(19.0), p	Effect size d_m	*EMM*	*EMM*	*EMM*	t(19.0), p	Effect size d_m	t(18.9), p	Effect size d_m
pNN50 [%]	13.06 [8.08, 18.03]	23.70 [14.72, 32.68]	22.12 [14.96, 29.27]	3.78, **.001****	1.05 [0.29, 1.81]	4.12, **< .001*****	0.89 [0.28, 1.51]	12.34 [6.46, 18.23]	21.51 [11.06, 31.97]	16.75 [8.38, 25.11]	2.82, **.011***	0.90 [0.11, 1.69]	1.73, .101	0.43 [0.13, 0.99]
SDNN [ms]	56.54 [46.50, 66.57]	86.85 [72.07, 101.63]	70.21 [59.63, 80.79]	5.35 **< .001*****	1.96 [0.76, 3.15]	3.40 **.003****	0.88 [0.20, 1.57]	51.32 [39.42, 63.23]	64.48 [47.20, 81.75]	57.62 [45.10, 70.14]	2.01 .059	0.85 [-0.12 1.82]	1.36 .191	0.41 [-0.25 1.06]
lnLF	8.05 [7.60, 8.49]	9.35 [8.89, 9.80]	7.96 [7.53, 8.39]	6.11, **< .001*****	2.41 [1.01, 3.80]	-0.59, .560	-0.16 [-0.74, 0.41]	7.57 [7.04, 8.09]	7.85 [7.32, 8.39]	7.50 [6.98, 8.01]	1.17, .258	0.53 [-0.45, 1.51]	-0.41, .686	-0.13 [-0.79, 0.53]
lnHF	7.05 [6.72, 7.38]	6.84 [6.39, 7.29]	7.26 [6.85, 7.67]	-1.27 .221	-0.39 [-1.07, 0.28]	1.51 .148	0.40 [-0.19, 0.99]	6.63 [6.24, 7.02]	7.07 [6.54, 7.60]	6.90 [6.42, 7.38]	2.35 **.030***	0.84 [-0.01 1.69]	1.66 .114	0.51 [-0.18, 1.19]
LF-norm	.48 [.41, .55]	.80 [0.73, 0.86]	.41 [.03, .48]	8.39 **< .001*****	2.81 [1.32, 4.31]	-2.62 **.017***	-0.59 [-1.13, -0.04]	.41 [.33, .49]	.48 [.40, .56]	.34 [.26, .42]	1.55 .137	0.60 [-0.26, 1.46]	-2.33 **.031***	-0.60 [-1.22, 0.01]

HF-norm	.20 [.15, .25]	.08 [.04, .11]	.25 [.17, .33]	-4.19 **< .001*****	-1.33 [-2.23, -0.42]	1.97 .064	0.60 [-0.10, 1.29]	.17 [.11, .23]	.24 [.20, .28]	.21 [.12, .31]	2.04 .055	0.75 [-0.09, 1.59]	1.29 .214	0.45 [-0.31, 1.21]
LF/HF	3.44 [2.16, 4.72]	14.89 [10.84, 18.94]	3.34 [1.80, 4.88]	5.33 **< .001*****	3.27 [1.27, 5.26]	-0.16 .876	-0.03 [-0.40, 0.35]	3.38 [1.87, 4.89]	3.66 [-1.02, 8.35]	2.80 [0.99, 4.60]	0.11 .910	0.08 [-1.40 1.56]	-0.80 .427	-0.17 [-0.61, 0.27]
PB-RSA	-5.39 [-5.73, -5.05]	-5.41 [-5.88, -4.94]	-5.17 [-5.57, -4.77]	-0.13 .901	-0.04 [-0.75, 0.67]	1.53 .143	0.44 [-0.20, 1.08]	-5.84 [-6.24, -5.44]	-5.29 [-5.84, -4.74]	-5.58 [-6.05, -5.11]	2.77 **.012***	1.08 [0.12 2.04]	1.52 .146	0.50 [-0.23, 1.24]

Table B2 – Between-Group Comparisons of Additional HRV Measures

Note. Comparisons are based on the differences of baseline adjusted estimated marginal means between groups (active control to biofeedback group). The 95% CIs are presented in square brackets. P values below .05 are displayed in bold font. Findings confirmed that the use of biofeedback for cardiac self-regulation training significantly increases vagally-mediated heart rate variability during and after training. HRV = heart rate variability; CI = confidence interval; pNN50 = percentage of successive normal heartbeat time intervals that differ by more than 50 ms; SDNN = standard deviation of successive normal heartbeat time intervals; lnLF = natural logarithm of absolute low frequency power of the heart rate signal; lnHF = natural logarithm of absolute high frequency power of the heart rate signal; LF-norm = relative power of the low frequency band of the heart rate signal; LF-norm = relative power of the high frequency band of the heart rate signal; LF/HF = absolute power ratio of the low and high frequency bands of the heart rate signal, PB-RSA = natural logarithm of the respiratory sinus arrhythmia calculated by the Porges-Bohrer method.
$^{***}p < .001$. $^{**}p < .01$. $^{*}p < .05$.

HRV measure	Training (n = 119)				Recovery (n = 119)			
	Difference	z ratio	*p*	Effect size d_m	Difference	z ratio	*p*	Effect size d_m
pNN50 [%]	1.61 [-2.92, 6.14]	0.70	.485	0.16 [0.29, 0.61]	4.41 [-0.12, 8.93]	1.91	.057	0.43 [0.05, 0.92]
SDNN [ms]	22.91 [14.89, 30.94]	5.60	**< .001*****	1.48 [0.65, 2.31]	11.59 [3.57, 19.61]	2.83	**.005****	0.75 [0.14, 1.36]
lnLF	1.37 [1.08, 1.67]	9.231	**<.001*****	2.55 [1.31, 3.78]	0.29 [0.001, 0.58]	1.967	**.049***	0.54 [0.05, 1.13]
lnHF	-0.37 [-0.63, -0.10]	-2.71	**.007****	-0.69 [-1.27, -0.11]	0.23 [-0.04, 0.50]	1.71	.087	0.44 [-0.10, 0.97]
LF-norm	.30 [.25, .35]	11.85	**< .001*****	2.70 [1.43, 3.96]	.05 [.00, .10]	1.96	.051	0.45 [-0.04, 0.93]
HF-norm	-.16 [-.21, -.12]	-6.80	**< .001*****	-1.78 [-2.71, -0.85]	.05 [.00, .10]	2.03	**.043***	0.53 [-0.03, 1.09]
LF/HF	11.27 [9.04, 13.50]	9.91	**< .001*****	3.22 [1.70, 4.76]	0.09 [-2.14, 2.32]	0.08	0.939	0.03 [-0.61, 0.66]
PB-RSA	-0.24 [-0.50, 0.02]	-1.81	.070	-0.47 [-1.02, 0.08]	0.29 [0.03, 0.55]	2.18	**.029***	0.57 [0.00, 1.12]

References

Laborde, S., Mosley, E., & Thayer, J. F. (2017). Heart Rate Variability and Cardiac Vagal Tone in Psychophysiological Research – Recommendations for Experiment Planning, Data Analysis, and Data Reporting. *Frontiers in Psychology*, *8*. https://doi.org/10.3389/fpsyg.2017.00213
