# Supplementary material for: Respiratory sinus arrhythmia during biofeedback is linked to persistent improvements in attention, short-term memory, and positive self-referential episodic memory
Source: Front Neurosci. 2022 Sep 13;16:791498. doi: 10.3389/fnins.2022.791498 (PMC9514056; doi:10.3389/fnins.2022.791498)
Supplement: Supplementary file 3 [file Data_Sheet_3.pdf]

Supplementary Material C

HRV Mediation Analysis

To check the influence of the respiration rate and heart rate on measures of HRV reported in Table 2 in the manuscript, a post-hoc mediation analysis was performed using the R-package lavaan (Rosseel, 2012). Direct and indirect effects were evaluated for temporal changes in P2T-RSA and RMSSD from the baseline to the training phase and from the baseline to the recovery phase. The model was defined as follows:

```
model <- ' # direct effect

            P2T-RSA (or RMSSD) ~ new BMI + b1*respiration rate + b2*heart rate + c*phase

          # mediator

            respiration rate ~ a1*phase

            heart rate ~ a2*phase

          # direct effect

            Direct := c

          # indirect effects (a*b)

            respiration rate := a1*b1

            heart rate := a2*b2

          # total effect

            total := c + (a1*b1) + (a2*b2)

fit <- sem(model, data)

summary(fit, standardized=TRUE)
```

Table C1 – Mediation effect of respiration rate and heart rate on temporal changes in HRV

Note. Indirect effects of the respiration rate and heart rate on changes in HRV (RMSSD and P2T-RSA) were checked by means of a mediation analysis. Effects were calculated across participants in respect to the temporal changes between measures taken at resting state before (baseline) and 5 min after training (recovery) as well as between measures taken at resting state and training. Latent and observed variables were standardized to obtain standardized parameters of the effect size. Respiration rate had the largest effect on P2T-RSA, especially for changes from baseline to training, but none on RMSSD. Conversely, RMSSD was not significantly affected by respiration rate. Heart rate had a mediating effect on both measures which was stronger in RMSSD. The relative effect of heart rate was specifically pronounced on changes between baseline and recovery. The results underline firstly that P2T-RSA reflects respiratory-linked cardiac vagal control opposed to RMSSD which is relatively unaffected by respiration. Secondly, the findings demonstrate that HRV scores were influenced by cycle length dependence (McCraty & Shaffer, 2015). HRV = heart rate variability; RMSSD = root mean square of successive heartbeat interval differences; P2T-RSA = natural logarithm of the respiratory sinus arrhythmia calculated by the peak-to-trough method.

	P2T-RSA				RMSSD			
Mediation effect	Estimate	*z*	*p*	Standardized	Estimate	*z*	*p*	Standardized
Baseline to Training								
Direct	0.17 (0.05)	3.133	**.002**	0.113	6.56 (2.51)	2.616	**.009**	0.152
Respiration rate	0.60 (0.06)	9.479	**<.001**	0.398	1.81 (1.49)	1.220	.223	0.042
Heart rate	0.10 (0.03)	3.569	**<.001**	0.067	6.26 (1.69)	3.701	**<.001**	0.145
Total	0.87 (0.08)	11.107	**<.001**	0.578	14.63 (2.62)	5.590	**<.001**	0.339
Baseline to Recovery								
Direct	0.05 (0.04)	1.144	.253	0.041	2.46 (1.75)	1.406	.160	0.069
Respiration rate	0.13 (0.05)	2.494	**.013**	0.110	0.74 (0.41)	1.821	.069	0.021
Heart rate	0.10 (0.02)	3.859	**<.001**	0.081	5.85 (1.46)	4.137	**<.001**	0.164
Total	0.27 (0.07)	3.755	**<.001**	0.232	9.05 (2.20)	4.120	**<.001**	0.253

References

Rosseel, Y. (2012). lavaan: An R Package for Structural Equation Modeling. *Journal of Statistical Software*, **48**(2), 1–36. https://doi.org/10.18637/jss.v048.i02.

McCraty, R., & Shaffer, F. (2015). Heart Rate Variability: New Perspectives on Physiological Mechanisms, Assessment of Self-regulatory Capacity, and Health risk. *Global Advances in Health and Medicine*, *4*(1), 46–61. https://doi.org/10.7453/gahmj.2014.073
