# Supplementary material for: Respiratory sinus arrhythmia during biofeedback is linked to persistent improvements in attention, short-term memory, and positive self-referential episodic memory
Source: Front Neurosci. 2022 Sep 13;16:791498. doi: 10.3389/fnins.2022.791498 (PMC9514056; doi:10.3389/fnins.2022.791498)
Supplement: Supplementary file 4 [file Data_Sheet_4.pdf]

Supplementary Material D

Episodic Memory Measures

1 Encoding Responses

Table D1 – Results of the Encoding Phase

Note. Average values of the number of responses and the reaction time were calculated for each cell (DV ~ group x test x encoding condition x valence). ANOVAs were performed for each encoding condition separately. Raw values and residuals for the encoding response reaction time were found not to be normally distributed. Therefore, the median and IQR are reported, and ANOVAs were based on values transformed with the natural logarithm. Sample variations (i.e., standard deviations and IQRs) are presented in parenthesis. The number of self-attributed adjectives or self-related autobiographical memories remained stable between groups and tests. On the contrary, group x test interaction effects were observed in the response reaction time at encoding. Hence the reaction time was added as a covariate to the statistical model designed for the memory performance analysis. ANOVA = analysis of variance, $\boldsymbol{\eta_p^2}$ = partial eta square; IQR = inter quartile range.
$^{***}p < .001$. $^{**}p < .01$. $^{*}p < .05$.

Encoding condition	Mean responses								Analyses of variance				
	Biofeedback group				Active control group				Main and interaction effects				
	Pre		Post		Pre		Post						
	Positive	Negative	Positive	Negative	Positive	Negative	Positive	Negative					
	**Number of responses during encoding** (max. value = 6, *N* = 352)												
	Mean (*SD*)								Group	Time	Valence	Group*Time	Group*Time* Valence
Perceptive: correct responses	5.58<br>(0.79)<br>*n* = 12	5.50<br>(1.17)<br>*n* = 12	4.08<br>(1.88)<br>*n* = 12	5.08<br>(1.17)<br>*n* = 12	4.70<br>(1.70)<br>*n* = 10	5.50<br>(0.85)<br>*n* = 10	4.20<br>(1.87)<br>*n* = 10	5.00<br>(1.25)<br>*n* = 10	*F*(1,80) = 0.51<br>*p* = .476<br>η_p^2 = 0.006<br>*ns*	*F*(1,80) = 6.03<br>*p* = .016<br>η_p^2 = 0.070<br>*	*F*(1,80) = 4.49<br>*p* = .037<br>η_p^2 = 0.053<br>*	*F*(1,80) = 0.60<br>*p* = .443<br>η_p^2 = 0.007<br>*ns*	*F*(1,80) = 0.83<br>*p* = .365<br>η_p^2 = 0.010<br>*ns*
Semantic: correct responses	5.67<br>(0.49)<br>*n* = 12	5.58<br>(0.67)<br>*n* = 12	4.33<br>(1.78)<br>*n* = 12	5.75<br>(0.62)<br>*n* = 12	4.900<br>(1.20)<br>*n* = 10	5.00<br>(0.94)<br>*n* = 10	4.40<br>(1.71)<br>*n* = 10	5.50<br>(1.08)<br>*n* = 10	*F*(1,80) = 2.46<br>*p* = .121<br>η_p^2 = 0.030<br>*ns*	*F*(1,80) = 1.42<br>*p* = .236<br>η_p^2 = 0.017<br>*ns*	*F*(1,80) = 6.71<br>*p* = .011<br>η_p^2 = 0.077<br>*	*F*(1,80) = 1.42<br>*p* = .236<br>η_p^2 = 0.017<br>*ns*	*F*(1,80) = 0.26<br>*p* = .611<br>η_p^2 = 0.003<br>*ns*
Semantic self-reference: self-attributed adjectives	4.83<br>(1.19)<br>*n* = 12	1.17<br>(0.94)<br>*n* = 12	4.25<br>(1.36)<br>*n* = 12	1.17<br>(1.12)<br>*n* = 12	5.10<br>(1.29)<br>*n* = 10	1.50<br>(1.08)<br>*n* = 10	4.90<br>(1.20)<br>*n* = 10	1.20<br>(0.92)<br>*n* = 10	*F*(1,80) = 1.71<br>*p* = .195<br>η_p^2 = 0.021<br>*ns*	*F*(1,80) = 1.22<br>*p* = .273<br>η_p^2 = 0.015<br>*ns*	*F*(1,80) = 205<br>*p* < .001<br>η_p^2 = 0.719<br>***	*F*(1,80) = 0.01<br>*p* = .933<br>η_p^2 <0.001<br>*ns*	*F*(1,80) = 0.48<br>*p* = .488<br>η_p^2 = 0.006<br>*ns*
Episodic self-reference: memories recalled	3.75<br>(1.55)<br>*n* = 12	2.67<br>(1.56)<br>*n* = 12	4.08<br>(1.56)<br>*n* = 12	2.25<br>(1.91)<br>*n* = 12	4.60<br>(1.51)<br>*n* = 10	2.50<br>(1.35)<br>*n* = 10	4.70<br>(1.77)<br>*n* = 10	2.10<br>(1.45)<br>*n* = 10	*F*(1,80) = 0.71<br>*p* = .403<br>η_p^2 = 0.009<br>*ns*	*F*(1,80) = 0.08<br>*p* = .780<br>η_p^2 <0.001<br>*ns*	*F*(1,80) = 31.0<br>*p* < .001<br>η_p^2 = 0.279<br>***	*F*(1,80) = 0.03<br>*p* = .875<br>η_p^2 <0.001<br>*ns*	*F*(1,80) = 0.03<br>*p* = .855<br>η_p^2 <0.001<br>*ns*
	**Encoding response reaction time** [ms] (*N* = 1053)												
	Median (*IQR*)								Group	Time	Valence	Group*Time	Group*Time* Valence
Perceptive	578<br>(402)<br>*n* = 36	672<br>(973)<br>*n* = 36	898<br>(1269)<br>*n* = 36	883<br>(1219)<br>*n* = 36	812<br>(1211)<br>*n* = 30	695<br>(1336)<br>*n* = 30	476<br>(445)<br>*n* = 30	313<br>(301)<br>*n* = 30	*F*(1,256) = 3.00<br>*p* = .084<br>η_p^2 = 0.012<br>*ns*	*F*(1,256) = 5.23<br>*p* = .023<br>η_p^2 = 0.020<br>*	*F*(1,256) = 0.19<br>*p* = .662<br>η_p^2 = 0.001<br>*ns*	*F*(1,256) = 12.93<br>*p* < .001<br>η_p^2 = 0.048<br>***	*F*(1,256) = 1.56<br>*p* = .213<br>η_p^2 = 0.006<br>*ns*
Semantic	484<br>(570)	508<br>(789)	625<br>(1179)	594<br>(937)	555<br>(547)	422<br>(512)	328<br>(308)	296<br>(93)	*F*(1,256) = 16.47	*F*(1,256) = 1.54	*F*(1,256) = 2.81	*F*(1,256) = 11.42	*F*(1,2576= 0.14

	n = 36	*n* = 36	*n* = 36	*n* = 36	*n* = 30	*n* = 30	*n* = 30	*n* = 30	$p < 0.001$<br>$\eta_p^2 = 0.060$<br>***	$p = .216$<br>$\eta_p^2 = 0.006$<br>*ns*	$p = .095$<br>$\eta_p^2 = 0.011$<br>*ns*	$p < .001$<br>$\eta_p^2 = 0.043$<br>***	$p = .709$<br>$\eta_p^2 < 0.001$<br>*ns*
Semantic self-reference	688<br>(770)<br>*n* = 36	562<br>(460)<br>*n* = 36	898<br>(1028)<br>*n* = 36	578<br>(946)<br>*n* = 35	454<br>(480)<br>*n* = 30	726<br>(864)<br>*n* = 30	312<br>(118)<br>*n* = 30	336<br>(362)<br>*n* = 30	$F(1, 255) =$<br>9.51<br>$p = .002$<br>$\eta_p^2 = 0.036$<br>**	$F(1, 255) =$<br>11.59<br>$p < .001$<br>$\eta_p^2 = 0.043$<br>***	$F(1, 255) =$<br>1.32<br>$p = .252$<br>$\eta_p^2 = 0.005$<br>*ns*	$F(1, 255) =$<br>12.26<br>$p < .001$<br>$\eta_p^2 = 0.046$<br>***	$F(1, 255) =$<br>0.58<br>$p = .448$<br>$\eta_p^2 = 0.002$<br>*ns*
Episodic self-reference	1578<br>(2172)<br>*n* = 36	1453<br>(1297)<br>*n* = 36	2086<br>(2890)<br>*n* = 36	1219<br>(1649)<br>*n* = 35	617<br>(4957)<br>*n* = 30	562<br>(2097)<br>*n* = 30	391<br>(297)<br>*n* = 29	453<br>(781)<br>*n* = 29	$F(1, 253) =$<br>18.45<br>$p < .001$<br>$\eta_p^2 = 0.068$<br>***	$F(1, 253) =$<br>9.90<br>$p = .002$<br>$\eta_p^2 = 0.038$<br>**	$F(1, 253) =$<br>0.37<br>$p = .545$<br>$\eta_p^2 = 0.001$<br>*ns*	$F(1, 253) =$<br>5.36<br>$p = .021$<br>$\eta_p^2 = 0.021$<br>*	$F(1, 256) =$<br>1.65<br>$p = .200$<br>$\eta_p^2 = 0.006$<br>*ns*

2 Self-Reference Effect

When the SRE was calculated as the memory score difference from the semantic to the self-semantic condition, the same trend as in the main results was observed. No statistically significant group x test x valence or group x test interaction effects were observed for any of the measures. Pre-test values per valence did not differ significantly between groups for any measure.

Table D2 – Self-Reference Effect (SRE)

Note. The SRE is defined here for each variable as the episodic memory score difference from the semantic to the self-semantic condition. EMMs of pre-to-post changes, their correlation (groups combined) with baseline-adjusted level of HRV during training, and the group x test interaction effect are presented for each memory variable. Statistics were derived from robust linear mixed-effect models. The behavior at encoding was controlled for. Standard deviations are presented in parenthesis. 95% CIs are presented in square brackets. EMMs in each group that exhibited significant pre-to-post-test differences and p values below .06 are displayed in bold font. Subjective and objective recollection reflect the proportions of correct remember responses and source recollections to studied items, respectively. The results reflect outcomes where the SRE is defined as the semantic-to-self-episodic difference (see Table 5), however, with less pronounced effects. EMM = estimated marginal means; ANCOVA = analysis of covariance; η_p^2 = partial eta square.

Variable	Pre-to-post change in SRE		ANCOVA: Group x Test Effect				Spearman correlation with P2T-RSA	
	Biofeedback Group (*n* = 12)	Active Control Group (*n* = 10)	*df*	*F*	*p*	η_p^2	*r*(19)	*p*
Negative items								
Free recall hit rate	-.08 (.09)	-.05 (.10)	1, 19.5	1.14	.298	0.06	-.25	.271
Subjective recollection hit rate	-.07 (.17)	.20 (.19)	1, 19.5	1.39	.253	0.07	-.01	.727
Objective recollection hit rate	.00 (.16)	.03 (.18)	1, 19.5	0.13	.736	0.00	.12	.608
Old/new discrimination (*d'*)	0.12 (0.23)	-0.30 (0.30)	1, 19.5	0.99	.333	0.05	.16	.483
Positive items								
Free recall hit rate	.15 (.09)	.15 (.10)	1, 19.2	0.00	.950	0.00	.24	.299
Subjective recollection hit rate	.08 (.17)	-.25 (.19)	1, 19.3	1.50	.236	0.07	.18	.427
Objective recollection hit rate	.25 (.16)	-.20 (.18)	1, 19.3	2.96	.101	0.13	.23	.317
Old/new discrimination (*d'*)	0.30 (0.28)	0.24 (0.30)	1, 19.2	0.01	.933	0.00	.13	.564
