# Supplementary material for: Respiratory sinus arrhythmia during biofeedback is linked to persistent improvements in attention, short-term memory, and positive self-referential episodic memory
Source: Front Neurosci. 2022 Sep 13;16:791498. doi: 10.3389/fnins.2022.791498 (PMC9514056; doi:10.3389/fnins.2022.791498)
Supplement: Supplementary file 5 [file Data_Sheet_5.pdf]

Supplementary Material E

Follow-Up Questionnaire

1 Questions in English

Q1: "Do you think the training program has been beneficial to your cognitive performance (memory, attention, short-term memory, planning skills, etc.)?"

Q2: "Have you experienced a noticeable effect on your daily life (health, emotions, performance, mindfulness, etc.) as a result of the training?"

Q3: "Was it easy for you to follow the training instructions?"

2 Original questions

Q1 : « Pensez-vous que le programme d'entraînement a aidé votre performance cognitive (mémoire, attention, mémoire à court terme, capacités de planification etc.) ? »

Q2 : « Avez-vous ressenti un effet perceptible sur votre vie quotidienne (santé, émotions, performance, pleine conscience, etc.) à travers l'entraînement ? »

Q3 : « A-t-il été facile pour vous de suivre les instructions de l'entraînement ? »

3 Results

Note. Q1, Q2, and Q3 were evaluated using a 5-point Likert scale from 1 (*no, not at all*) to 5 (*yes, absolutely*). Original scale: 1: *No, pas de tout*; 2: *Plutôt non*; 3: *Pas sûre*; 4: *Plutôt oui*; 5: *Oui, tout à fait.* Group differences were calculated using the nonparametric Mann-Whitney U Test. The test was two-tailed and was performed with a significance level of alpha = .05.

Question	Biofeedback group (Group A, n = 8)		Active control group (Group B, n = 7)		Group difference		
	M	*SD*	*M*	*SD*	*U*	*z*	*p*
Q1	3.50	0.87	3.71	1.28	24.5	-0.35	.726
Q2	3.25	0.97	2.57	1.40	19	0.98	.327
Q3	4.38	0.99	4.29	0.70	23.5	0.46	.646
